# Repeated Treadmill Run Preconditioning Induces Prolonged Attenuation of Craniofacial Pain-like Behaviors and Changes in Brain Responses Associated with Persistent Craniofacial Inflammation in Male Mice

**DOI:** 10.3390/biomedicines14071576

**Published:** 2026-07-14

**Authors:** Andi Sitti Hajrah Yusuf, Mana Hasegawa, Yuya Iwamoto, Takumi Kato, Aditya Anugrah, Yoshito Kakihara, Kensuke Yamamura, Keiichiro Okamoto

**Affiliations:** 1 Division of Oral Physiology, Faculty of Dentistry, Niigata University Graduate School of Medicine, Dentistry and Health Sciences, Niigata 951-8514, Japan; hajrahyusuf@dent.niigata-u.ac.jp (A.S.H.Y.); mana@dent.niigata-u.ac.jp (M.H.); yiwa@dent.niigata-u.ac.jp (Y.I.); ktakumi@dent.niigata-u.ac.jp (T.K.); n25d356e@mail.cc.niigata-u.ac.jp (A.A.); yamamurak@dent.niigata-u.ac.jp (K.Y.); 2 Department of Oral and Maxillofacial Surgery, Faculty of Dentistry, Hasanuddin University, Makassar 90245, Indonesia; 3 Division of General Dentistry and Dental Clinical Education Unit, Niigata University Medical and Dental Hospital, Niigata 951-8514, Japan; 4 Division of Dental Clinical Education, Faculty of Dentistry, Niigata University Graduate School of Medicine, Dentistry and Health Sciences, Niigata 951-8514, Japan; 5 Department of Oral Biology, Faculty of Dental Medicine, Airlangga University, Surabaya 60132, Indonesia; 6 Division of Dental Pharmacology, Faculty of Dentistry, Niigata University Graduate School of Medicine, Dentistry and Health Sciences, Niigata 951-8514, Japan; kakihara@dent.niigata-u.ac.jp; 7 Sakeology Center, Niigata University, Niigata 950-2181, Japan

**Keywords:** pain, craniofacial inflammation, treadmill run preconditioning, epigenetic change, neural activity

## Abstract

**Background/Objectives**: Regular physical exercise conditioning attenuates nociceptive responses. However, it remains unclear whether physical exercise performed before local inflammation exerts prolonged preventive effects. This study determined whether treadmill run (TR) preconditioning produces sustained preventive effects on craniofacial nociception and associated brain responses following persistent craniofacial inflammation. **Methods**: Male C57BL/6J mice were assigned to sedentary or TR groups. Daily TR conditioning was performed for 10 days before masseter muscle injection of complete Freund’s adjuvant (CFA) on Day 0. Craniofacial-pain- and related anxiety-like behaviors were determined by the orofacial formalin, elevated plus maze, and open-field tests before and 3 (CFA3) or 7 (CFA7) days after CFA injection. Brain responses in the amygdala, insular cortex, hippocampal CA1, and primary motor cortex were assessed using multiple epigenetic- and neural-activity-related markers. **Results**: Under sedentary conditions, both CFA3 and CFA7 groups showed increased pain- and anxiety-like behaviors and elevated expression of epigenetic- and neural-activity-related markers in most brain regions. TR preconditioning attenuated these behavioral responses even three and seven days after TR cessation and altered the expression of epigenetic markers in several brain regions, although the direction of change varied by region and time point. TR preconditioning consistently reduced the expression of neural activity markers in most brain areas in both the CFA3 and CFA7 groups, with a few exceptions. **Conclusions**: TR preconditioning exerted prolonged preventive effects on craniofacial-pain-like behaviors and associated brain responses following craniofacial inflammation.

## 1. Introduction

Regular physical activity is recognized to influence both physiological and psychological functions, including pain processing [1,2]. In both clinical and preclinical studies, physical exercise has been reported to attenuate pain-related behaviors and modulate brain responses under various pathological conditions [3,4]. In particular, repeated physical exercise might induce persistent neural changes in the brain that could alter subsequent responses to nociceptive stimuli [5,6,7].

Among physical exercise paradigms, repeated treadmill run (TR) has been widely used in experimental animal studies to investigate the effects of physical conditioning on pain-related responses [8]. Previous studies have demonstrated that regular TR attenuates craniofacial-pain- and anxiety-like behaviors [9,10].

In clinical settings, however, individuals experiencing ongoing pain are often unable to perform sufficient physical activity because pain itself can interfere with exercise participation [11]. Therefore, understanding whether daily physical exercise conditioning performed before the onset of painful conditions modifies subsequent pain-related responses might have practical relevance. This study suggests that repeated physical exercise prior to nociceptive events might function as a form of preconditioning that alters later behavioral and brain responses associated with nociception.

Inflammatory pain is accompanied not only by enhanced nociceptive responses but also by alterations in emotional and brain responses [12,13]. In the present study, we employed a complete Freund’s adjuvant (CFA)-induced model of persistent craniofacial inflammation [14]. This model has been shown to induce prolonged behavioral changes related to craniofacial nociception together with changes in brain-response-related markers involved in trigeminal nociceptive and affective processing for at least one week [15,16]. However, it remains unclear whether TR preconditioning is sufficient to attenuate craniofacial-pain- and related anxiety-like behaviors under conditions of CFA-evoked craniofacial inflammation, even after the cessation of TR.

To investigate brain responses potentially associated with the prolonged effects of TR preconditioning, immunohistochemical analyses were conducted using markers of epigenetic regulation as well as markers of neural activity [17,18,19,20] in brain regions associated with craniofacial nociception, including the amygdala [21], insular cortex [16], and hippocampal CA1 [17]. Although the amygdala and hippocampal CA1 are classically associated with emotion and memory, accumulating evidence suggests that these regions are also involved in chronic pain processing [22,23]. Consistent with this, our previous studies demonstrated that repeated TR modulated brain responses in cortical regions, including the insular cortex, under conditions of psychological stress [9,10]. However, the responses of these regions associated with inflammatory craniofacial pain remain unclear [16,24]. In addition, the primary motor cortex (M1) was included as a region of interest because of its role in motor function and its potential involvement in exercise-related neural responses associated with nociception [25,26].

The present experimental design aims to demonstrate the preventive effects of 10 days of repeated TR preconditioning on craniofacial-pain-like behaviors and brain responses during CFA-evoked persistent craniofacial inflammation, even after the cessation of TR.

## 2. Materials and Methods

### 2.1. Animals and Housing

Our study used 186 male C57BL/6J mice (Charles River Laboratories, Yokohama, Japan). All experimental procedures were reviewed and approved by the Intramural Animal Care and Veterinary Science Committee of Niigata University (permit number: SA01380). All procedures were conducted in accordance with institutional guidelines and complied with the Animal Research: Reporting In Vivo Experiments (ARRIVE) guideline 2.0 [27]. All efforts were made to minimize animal suffering and to use the minimum number of animals necessary to achieve the scientific objectives of the study. Animals were monitored daily for signs of distress, including body weight loss and abnormal posture. Humane endpoints were predefined as more than 10% body weight loss or signs of severe distress. Animals reaching these criteria would be euthanized immediately upon detection with an overdose of a 3-anesthetic mix (medetomidine, midazolam, and butorphanol; three times the standard anesthetic dose described in the later section).

No animals met the predefined humane endpoint criteria prior to scheduled euthanasia, and no animals were found dead during the experimental period. All animals were euthanized at the planned experimental endpoints according to the study design. All experimental procedures were performed by personnel who had completed institutional education and training for animal experimentation and were certified to conduct animal experiments in accordance with institutional regulations.

Upon arrival, C57BL/6J mice were six weeks old, weighing 19–21 g. The mice were randomly assigned to cages (four per clear acrylic cage, 30 × 20 × 15 cm) with ad libitum access to standard pellet food and water. All mice were acclimated to the housing environment for at least one week prior to the experiment. The housing facility was maintained under controlled temperature and humidity conditions, with a 12 h light/dark cycle (lights on: 07:00–19:00). The number of animals used is detailed in Supplemental Table S1.

### 2.2. Experimental Schedule and Groups

**Figure 1:**
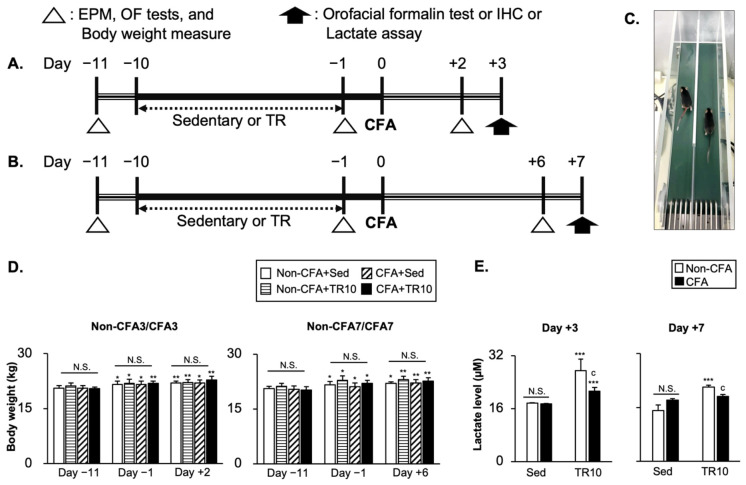
Experimental design. Mice were subjected to TR preconditioning or sedentary conditions from Day −10 to Day −1, followed by CFA injection on Day 0. Schematic diagrams of the experimental protocols for Day +3 (**A**) and Day +7 (**B**). Anxiety-like behaviors and body weight (white triangles) were assessed before CFA injection and on Day +2 (**A**) or Day +6 (**B**). Afterward, the mice were sacrificed for immunohistochemical analysis or a lactate assay (black arrows) on Day +3 or Day +7. In a separate group of animals, mice were subjected exclusively to the orofacial formalin test on Day +3 (**A**) or Day +7 (**B**) (black arrows). (**C**) The motorized treadmill used for rodents. (**D**) Body weight changes in all groups under sedentary or TR10 conditions. (**E**) Brain lactate levels measured on Day +3 and Day +7 in non-CFA and CFA mice under sedentary or TR10 conditions. *, *p* < 0.05; **, *p* < 0.001, ***, *p* < 0.0001 versus before TR (Day −11) or the corresponding sedentary group; c, *p* < 0.0001 versus the corresponding non-CFA group. N.S., not significant.

Mice underwent daily TR or sedentary conditioning for 10 consecutive days prior to the masseter muscle injection of CFA (CFA group) (Figure 1A,B).

The first aim of this study was to determine whether TR preconditioning (TR10) attenuates anxiety- and craniofacial-pain-like behaviors in mice with persistent craniofacial inflammation, even three or seven days after cessation of TR conditioning. Mice were subjected to anxiety-like behavioral assessments using the elevated plus maze and open-field tests, followed by immunohistochemistry on Day +3 or Day +7 (Figure 1A,B). Sedentary or TR conditioning was performed from Day −10 to Day −1. CFA was administered into the masseter muscle on Day 0. Body weight changes measured at all three time points (pre-TR, post-TR, and post-CFA injection) are presented in Figure 1D.

The second aim was to determine whether TR10 exerts lasting effects on brain responses. Brain responses were evaluated through epigenetic changes and neural activity, indicated by specific markers. Epigenetic changes were assessed via immunoreactivity for histone H3 acetylation, histone deacetylase (HDAC)1, and HDAC2. Neural activity was evaluated by immunoreactivity for phosphorylated cAMP response element-binding protein (pCREB), FosB, and c-Fos in selected brain regions. The use of multiple markers might provide a more reliable and biologically relevant interpretation than reliance on a single marker alone because certain neural changes might not be detectable by one marker, depending on the timing and/or nature of the neuronal response [21,22,23].

Following the completion of anxiety behavioral testing, animals were sacrificed for immunohistochemistry on Day 3 or Day 7 (Supplemental Table S1). The craniofacial-pain-like behaviors were examined in a separate experiment using the orofacial formalin test on Day 3 or Day 7. These mice were used exclusively for formalin-evoked pain assessment and were not included in the immunohistochemistry analyses (Supplemental Table S1). Additionally, information regarding the equipment used for TR and behavioral testing, as well as the reagents employed, including the manufacturer’s details (company, catalog number (Cat#), research resource identifiers (RRIDs), city, and country), is summarized in Supplemental Table S2.

### 2.3. TR Preconditioning

The TR conditionings were conducted daily from Day −10 to Day −1 in the morning (9:00 am) (Figure 1). The procedure has been outlined in our earlier publications [9,10]. Briefly, mice engaged in a 30 min exercise session on a motorized treadmill set to a speed of 6 m per minute (m/min), with no incline (Figure 1C). This relatively low-intensity TR protocol was selected based on previous preclinical studies showing that mild or regular aerobic exercise can attenuate pain- and anxiety-like behavioral responses in preclinical pain models [28,29]. In addition, our previous craniofacial-pain-related studies using the same TR paradigm demonstrated that repeated TR at 6 m/min for 30 min/day attenuated craniofacial-pain- and anxiety-like behavioral responses [9,10].

Mice assigned to the sedentary group were positioned in the treadmill apparatus for a duration of 30 min without engaging in running activity. After each TR session, the mice were returned to their home cages, and the treadmill lane was cleaned with 70% ethanol, wiped, and air-dried before the next use.

### 2.4. Craniofacial Inflammatory Model (CFA Group)

Mice were anesthetized via intraperitoneal injection of a mixed anesthetic cocktail consisting of 0.3 mg/kg medetomidine, 4.0 mg/kg midazolam, and 5.0 mg/kg butorphanol. The CFA (Mycobacterium tuberculosis, Supplemental Table S2), suspended in an oil/saline mixture (0.9% sodium chloride) at a 1:1 ratio, was used as the inflammatory agent. A 5 µL volume of CFA was injected into the left masseter muscle using a Hamilton syringe [15]. These mice were designated as the CFA group, whereas mice that did not receive any drug treatment in the craniofacial region constituted the non-CFA group. Following CFA injection, mice were maintained for either three days (CFA3 group) or seven days (CFA7 group). For the non-CFA groups, assessments were performed at the same time points as in the CFA groups, and they were designated as the non-CFA3 and non-CFA7 groups, respectively. At the time of CFA administration (Day 0), mice in all groups were randomly assigned to cages.

### 2.5. Anxiety-like Behavioral Test

Behavioral tests using the elevated plus maze [30,31] and open-field [32,33] tests were conducted before the start of TR preconditioning (Day −11), after a 10-day TR (Day −1), and 2 or 6 days after CFA injection (between 7 and 8 AM). The mice underwent a one-hour acclimatization period in the testing room prior to the commencement of the experiment. The experimenter conducting the study was blinded to the treatment conditions.

#### 2.5.1. Elevated Plus Maze Test

The experimental setup involved positioning a mouse at the center of an elevated maze, which was approximately 50 cm above the ground. The maze comprised two open arms and two closed arms. Time spent in the open arms was recorded over a 5 min observation period for subsequent analysis. The procedures for these are similar to those in our previous reports [10,15].

#### 2.5.2. Open-Field Test

Mice were placed individually in a clear acrylic cage with a black-frosted Plexiglas floor (45 × 45 × 30 cm, Supplemental Table S2) for 5 min. Locomotor activity was evaluated by monitoring movement across the field using computer-connected digital counters with infrared sensors. The total movement distance and the time spent in the center area were recorded over the 5 min observation period. Because locomotor activity was measured based on crossings of infrared sensor beams, the recorded values reflected relative movement activity rather than the actual physical distance traveled by the mice.

### 2.6. Craniofacial Pain-like Behavioral Test

The orofacial formalin test was performed on Day +3 or Day +7 to measure craniofacial-pain-like behaviors [34]. Briefly, a Hamilton syringe was used to inject 2 µL of 2.5% formalin, dissolved in 0.9% saline, into the central portion of the left masseter muscle. During this procedure, the animals were gently restrained with a soft plastic net for a duration not exceeding 5 s. Subsequently, the mice were placed back in their observation cage. The researchers then documented the duration of grooming or rubbing behavior on the side corresponding to the injection. Behavioral observations were conducted using a stopwatch across ten consecutive intervals, each lasting three minutes. The data analysis aimed to evaluate the total duration of the behavior, distinguishing between the early phase (0–9 min) and the later phase (10–30 min). The experimenter conducting the study was blinded to the treatment conditions.

### 2.7. Immunohistochemistry

#### 2.7.1. Preparations

After the completion of anxiety-like behavioral testing, mice were sacrificed on Day +3 or Day +7. All mice were subjected to deep anesthesia through the administration of the previously mentioned three mixed agents. Adequate depth of anesthesia was confirmed by the absence of pedal and palpebral reflexes prior to the procedure. Animals were euthanized through a process involving transcardial perfusion. Initially, 20 mL of saline was administered via the heart, followed by the introduction of 20 mL of cold 4% paraformaldehyde in 0.1 M phosphate-buffered saline (PBS, pH 7.4). To identify the left and right sides of the brain, a slight mark was made on the left cerebral cortex at a site unrelated to and distant from the regions of interest for subsequent analysis after perfusion. The brains, including the amygdala, insular cortex, hippocampal CA1, and primary motor cortex, were excised and subsequently immersed in a 4% paraformaldehyde solution for several days. Following this process, they were placed in a 30% sucrose solution in 0.1 M PBS and stored at 4 °C overnight. The brains were then sectioned transversely into 40 µm slices with a freezing microtome. Sections were collected from five wells containing cold 0.1 M PBS and transferred serially to multi-well tissue culture plates containing cold 0.1 M PBS, which were then used for immunohistochemistry.

#### 2.7.2. Immunostaining

Following multiple rinses with PBS, the sections were incubated overnight at room temperature with various antibodies, including affinity-purified rabbit histone H3 acetylation polyclonal antibody (1:2000), HDAC1 polyclonal antibody (1:4000), HDAC2 monoclonal antibody (1:4000), phospho-CREB polyclonal antibody (1:4000), FosB monoclonal antibody (1:2000), or c-Fos polyclonal antibody (1:1000). These antibodies were prepared in PBS containing Tween 20 (PBST) and 5% normal goat serum (NGS). After the incubation, the sections were rinsed twice with PBS and then exposed to a biotinylated goat anti-rabbit IgG antibody (1:200) in PBST with 5% NGS for 2 h at room temperature. Subsequently, the sections were washed twice with PBS, followed by the addition of an avidin–biotin–peroxidase complex for 1 h. The sections were then washed twice with Tris-buffered saline (TBS) and incubated in a diaminobenzidine and nickel solution, activated by 0.01% peroxidase. Upon visualization of immunoreactivity, the sections were washed with TBS, mounted on glass slides, and dried. The slides were then dehydrated in ethanol, cleared in xylene, and coverslipped. Notably, specific immunostaining was absent when each primary antibody was omitted.

#### 2.7.3. Quantifications

Brain sections were delineated according to landmark structures at each level, as outlined in the Franklin and Paxinos atlas (3rd edition) [35]. Reference points were determined by measuring specific distances from bregma in the rostral direction: basolateral (BLA) and central (CeA) portions of the amygdala (−1.22 mm to −1.34 mm), the insular cortex (+0.14 mm to −0.58 mm), anterior portion of the dorsal (dr) CA1 of the hippocampus (−1.46 mm to −1.70 mm, anterior dr-CA1), posterior portion of the dr and ventral (vl) CA1 of the hippocampus (posterior dr- and vl-CA1, −3.08 mm to −3.16 mm), and M1 (+0.98 to −0.46 mm). The investigators who photographed the brain sections (HJ, TK, YI, AD) were different from the person who examined the sections and quantified the positive cells using ImageJ (version 1.53; National Institutes of Health, Bethesda, MD, USA). The person performing the ImageJ analysis (MH, KO) was blinded to the experimental groups.

Microscopic images of each section were captured under identical conditions at ×10 magnification for the amygdala and M1 and at ×20 magnification for the insular cortex and hippocampal CA1. Two to three sections per region were analyzed, depending on tissue integrity; the mean per animal was used for statistics. Cell counts were conducted bilaterally in two subregions of the amygdala (0.3 × 0.3 mm) [36], insular cortex (0.7 × 1.0 mm) [19], three subregions of hippocampal CA1 (0.1 × 0.5 mm) [37], and M1 (0.7 × 1.0 mm) [38] using ImageJ (National Institutes of Health, Bethesda, MD, USA).

Images were converted to an eight-bit grayscale. The threshold value was determined based on preliminary analyses and was applied uniformly across all sections within each marker to ensure consistency. Histone H3 acetylation-, HDAC1-, HDAC2-, pCREB-, FosB-, and c-Fos-positive nuclei were considered to be positive signals showing grayscale contrast levels over 125 units (total possible range was 0–255) to eliminate very lightly stained nuclei constitutively present in the brain [39]. Cells were then counted automatically, with those exceeding 40 pixels being defined as cells within the region of interest [40]. The sample size in each brain area and treatment is presented in Supplemental Table S1B.

The results indicate the number of cells quantified independently for the ipsilateral side (left, CFA injection side) and the contralateral side (right) of each region. Small lesions were made in the cortical area outside the regions used for cell quantification using a surgical blade.

### 2.8. Measurement of the Brain Lactate Level

The lactate level in the brain was analyzed to assess the effects of TR preconditioning on brain metabolism under a persistent craniofacial inflammatory condition. Lactate is a major product of physical exercise [41], while repeated physical exercise increases the lactate levels in the blood serum and brain [42,43]. Mice were assigned to non-CFA or CFA-treated groups under either sedentary or TR preconditioning conditions to investigate the possible effects of TR on brain metabolism. Animals were sacrificed on Day +3 or Day +7 in both the non-CFA and CFA groups. The number of animals used for this study is presented in Supplemental Table S1A. Mice were transcardially perfused with saline (20 mL) under general anesthesia as described before, and the brains were collected for lactate analysis. Brain tissue blocks containing the bilateral amygdala, insular cortex, the anterior and posterior CA1 regions, and M1 areas were dissected and processed as a single sample (+1.0 to −3.5 mm rostral to bregma).

The lactate level in the brain tissue was measured using a colorimetric lactate assay kit (Supplemental Table S2), according to the manufacturer’s instructions. In brief, brain tissues from each experimental group were rapidly dissected, homogenized in ice-cold assay buffer, and centrifuged at 13,000 rpm for 10 min. The resulting supernatants and lactate standards were added to a 96-well plate and incubated with the working solution at 37 °C for 30 min. The colorimetric reaction product was measured by absorbance at 450 nm using a microplate reader. All samples were assayed in triplicate, and lactate concentrations were calculated from the standard curve.

### 2.9. Statistical Analysis

Statistical analyses were conducted using SPSS Statistics (version 21.0; IBM, Armonk, NY, USA). All data are presented as the mean ± standard deviation (SD). A statistical level of *p* < 0.05 was considered significant.

Anxiety-related behavior and body weight data were analyzed using a two-way repeated-measures analysis of variance (ANOVA) with time (Days −11, −1, and +2 or +6) as a within-subject factor and treatment (CFA condition × TR preconditioning) as a between-subject factor. Post hoc comparisons were performed using the Bonferroni test.

The behaviors indicative of craniofacial pain, measured by the duration of grooming or rubbing in both the early and late phases, were analyzed for each phase independently using a one-way ANOVA to assess differences among the treatment groups. Subsequent post hoc comparisons were conducted using the Bonferroni test.

Immunohistochemistry data were analyzed using two-way ANOVA, followed by the Bonferroni post hoc test for group comparisons. The number of immunopositive cells was evaluated on the ipsilateral and contralateral sides. The lactate level data from the CFA3 and CFA7 groups were separately analyzed using one-way ANOVA. Post hoc comparisons were performed using the Bonferroni test.

Spearman’s rank correlation analysis was performed to assess the relationships between formalin-evoked pain-like behavioral measures and the number of immune-positive cells for each marker (histone H3 acetylation, pCREB, FosB, and c-Fos) on the ipsilateral side of each brain region. Analyses were conducted separately within the non-CFA3, non-CFA7, CFA3, and CFA7 groups. Craniofacial-pain-like behaviors were quantified as the total duration of grooming and rubbing during a 30 min observation period following formalin injection. The sample size for this analysis is presented in Supplemental Table S1C.

## 3. Results

### 3.1. Body Weight (Figure 1D)

Body weight changes were compared before TR (Day −11), after TR (Day −1), and after CFA injection (Day +2 or Day +6). Two-way ANOVA revealed a significant main effect of time course (F(2, 72) = 99.74, *p* < 0.0001), but no significant main effect of group (F(3, 36) = 0.81, *p* = 0.446). Post hoc analysis demonstrated that all animals, regardless of treatment, showed similar significant increases in body weight over time.

### 3.2. Lactate Level (Figure 1E)

Brain lactate levels were measured 3 days (Day +3) and 7 days (Day +7) after cessation of the TR protocols. On Day +3, the lactate level was significantly higher in the TR10 group than in the corresponding sedentary non-CFA3 and CFA3 groups (both, *p* < 0.0001). Group comparisons within the TR10 group revealed that the lactate level in the CFA3 group was significantly lower than that in the non-CFA3 group (*p* < 0.0001).

On Day +7, under sedentary conditions, the lactate level in the CFA7 group was similar to that in the non-CFA7 group. In the TR10 group, the lactate level in the non-CFA7 group was significantly higher than that in the corresponding sedentary group (*p* < 0.0001), while the level in the CFA7–TR10 group was significantly lower than that in the non-CFA7–TR10 group (*p* < 0.0001).

### 3.3. TR Preconditioning Attenuates Anxiety-like Behavior

Anxiety-like behavioral data from Day +2 (CFA3) and Day +6 (CFA7), each including sedentary and TR10 mice and their corresponding non-CFA groups, are presented.

#### 3.3.1. Elevated Plus Maze Test

**Figure 2:**
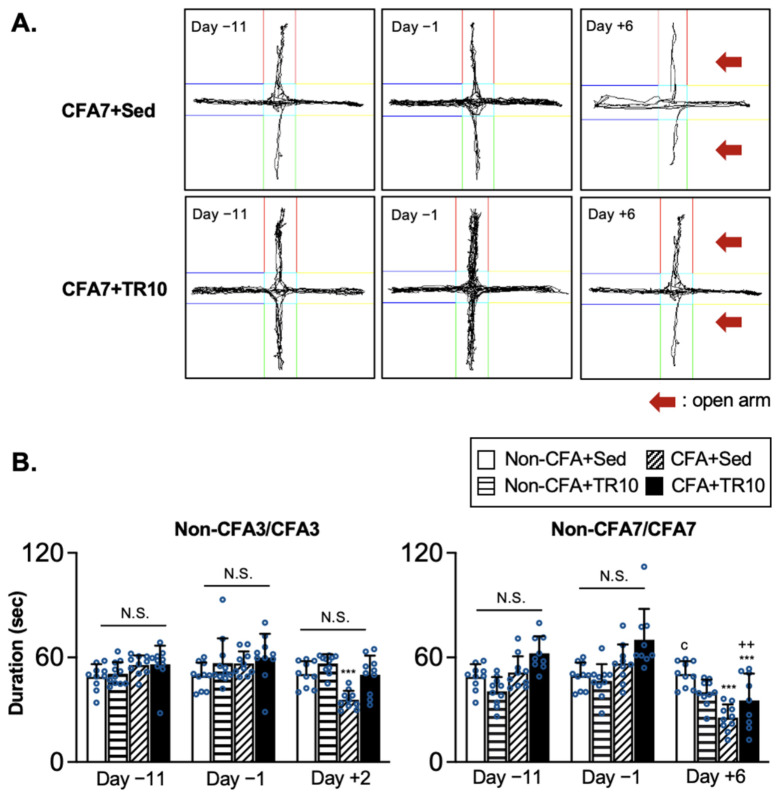
Effect of TR preconditioning on anxiety-like behaviors in the elevated plus maze test. (**A**) Representative tracking traces before (Day −11) and after TR (Day −1) and after CFA injection (Day +6) in the CFA7–sedentary and CFA7–TR10 groups. (**B**) Quantification of the time spent in the open arms (red arrows in (**A**)) for each group. ***, *p* < 0.0001 versus before TR within the same treatment group; c, *p* < 0.0001 versus the other three groups; ++, *p* < 0.0001 versus the CFA7–sedentary group. Open circles indicate individual data points. N.S., not significant.

Figure 2A shows representative trajectories of sedentary and TR10 mice in the CFA7 group during the elevated plus maze test, shown before (Day −11) and after TR (Day −1) and after CFA injection (Day +6).

In the CFA3 group, significant main effects of time course were observed (F(2, 111) = 17.26, *p* < 0.0001), whereas the main effect of treatment was not significant (F(3, 37) = 2.35, *p* < 0.09). The post hoc test revealed that time spent in the open arms of the CFA3–sedentary group on Day +2 (after CFA) was significantly shorter than on Day −11 (Figure 2B, left panel).

In the CFA7 group, significant main effects of both time course (F(2, 111) = 20.89, *p* < 0.0001) and treatment (F(3, 35) = 4.63, *p* < 0.008) were observed. Post hoc analysis revealed that time spent in the open arms in the CFA7–sedentary and CFA7–TR10 groups on Day +6 was significantly shorter than that on Day −11 (*p* < 0.0001, Figure 2B, right panel).

In the non-CFA7 and CFA7 groups (Figure 2B, right panel), comparisons among the four groups revealed that on Day +6, the non-CFA7–sedentary group spent significantly more time in the open arms than the other three groups (*p* < 0.0001), whereas the CFA7–TR10 group spent a significantly longer time in the open arms than the CFA7–sedentary group (*p* < 0.0001).

#### 3.3.2. Open-Field Test

**Figure 3:**
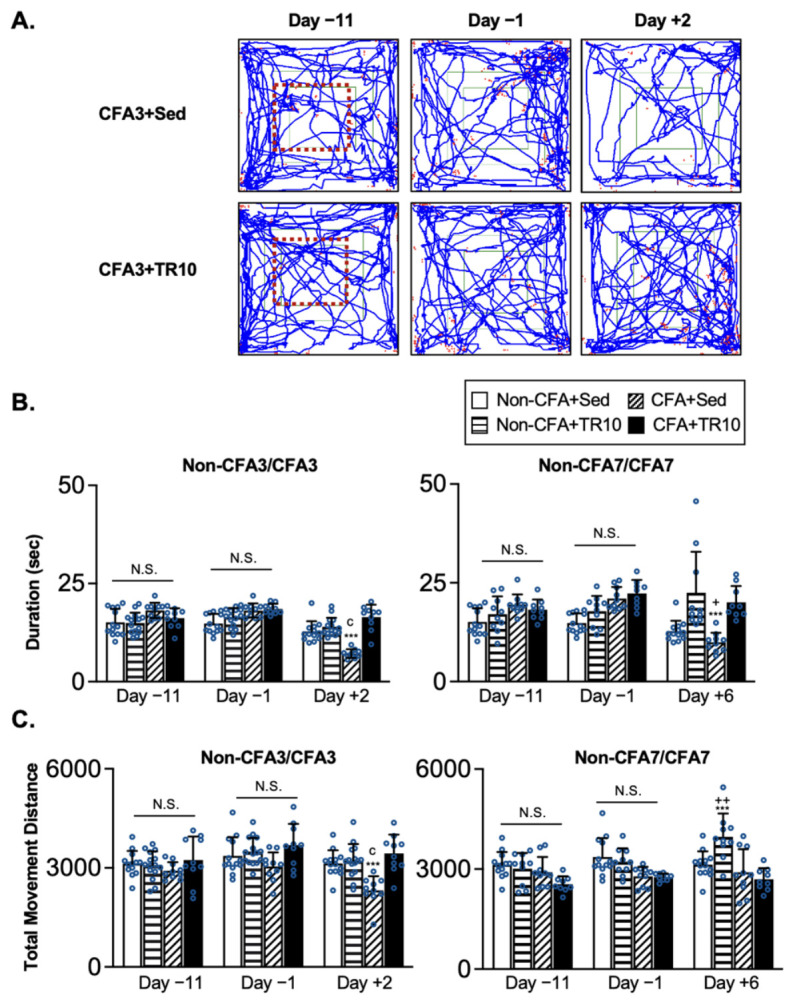
Effect of TR preconditioning on anxiety-like behaviors in the open-field test. (**A**) Representative tracking trajectory showing locomotor activities of sedentary and TR10 groups. (**B**) Quantification of the time spent in the center area (boxed area) in all groups. (**C**) Quantification of total movement distance in all groups. ***, *p* < 0.0001 versus before TR within the same treatment group; c, *p* < 0.0001 versus the other three groups; +, *p* < 0.003 versus the CFA7–TR10 group; ++, *p* < 0.0001 vs. the non-CFA7–sedentary group. Open circles indicate individual data points. N.S., not significant.

Figure 3A shows representative trajectories of sedentary and TR10 mice in the CFA3 group during the open-field test, recorded before (Day −11) and after TR (Day −1) and after CFA injection (Day +2).

- Time spent in the center area

Figure 3B (left panel) shows that both the non-CFA3 and CFA3 groups had significant main effects of time course (F(2, 88) = 43.9, *p* < 0.0001) and treatment (F(3, 44) = 8.6, *p* < 0.0001). The post hoc test revealed that time spent in the center area in the CFA3–sedentary group on Day +2 was significantly shorter than on Day −11 (*p* < 0.0001). Group comparisons on Day +2 revealed that time spent in the center area in the CFA3–sedentary group was significantly shorter than that in the other three groups (*p* < 0.0001).

In the non-CFA7 and CFA7 groups (Figure 3B, right panel), significant main effects of time course (F(2, 72) = 3.81, *p* < 0.027) and treatment (F(3, 38) = 9.3, *p* < 0.0001) were observed. The post hoc test revealed that time spent in the center area in the CFA7–sedentary group on Day +6 was significantly shorter than on Day −11 (*p* < 0.0001). On Day +6, group comparisons revealed that time spent in the center area in the CFA7–sedentary group was significantly shorter than that in the CFA7–TR10 group (*p* < 0.003).

b. Total movement distance

Figure 3C (left panel) shows that both the non-CFA3 and CFA3 groups had significant main effects of time course (F(2, 90) = 9.7, *p* < 0.0001) and treatment (F(3, 44) = 8.2, *p* < 0.0001). The post hoc test revealed that the total movement distance of the CFA3–sedentary group on Day +2 was significantly smaller than that on Day −11 (*p* < 0.0001). Group comparisons on Day +2 revealed that the total movement distance of the CFA3–sedentary group was significantly smaller than that of other groups (*p* < 0.0001).

In the non-CFA7 and CFA7 groups (Figure 3C, right panel), significant main effects of time course (F(2, 76) = 4.4, *p* < 0.02) and treatment (F(3, 38) = 13.1, *p* < 0.0001) were observed. The post hoc test revealed that the total movement distance of the non-CFA7–TR10 group on Day +6 was significantly greater than that on Day −11 (*p* < 0.0001), whereas the CFA7–sedentary group on Day +6 did not significantly differ among Days −11, −1, and +6 (*p* > 0.1). Group comparisons on Day +6 revealed that total movement distance in the non-CFA7–TR10 group was significantly greater than in the non-CFA7–sedentary group (*p* < 0.0001).

### 3.4. TR Preconditioning Attenuates Late-Phase Craniofacial Pain-like Behavior

The orofacial formalin test revealed a significant main effect of the experimental group in both the early (F(7, 72) = 16.3, *p* < 0.0001) and late (F(7, 72) = 26.3, *p* < 0.0001) phases. Results for the early and late phases are presented separately below.

#### 3.4.1. Early Phase (Figure 4A)

Post hoc analyses demonstrated that craniofacial-pain-like behavior durations were significantly greater in the CFA3 (*p* < 0.0001 and *p* < 0.001) and CFA7 (*p* < 0.004 and *p* < 0.001) groups compared with the corresponding non-CFA3 and non-CFA7 groups under both sedentary and TR10 conditions. However, no significant differences were found between the sedentary and TR10 groups within the CFA3 or CFA7 group (*p* > 0.1).

**Figure 4:**
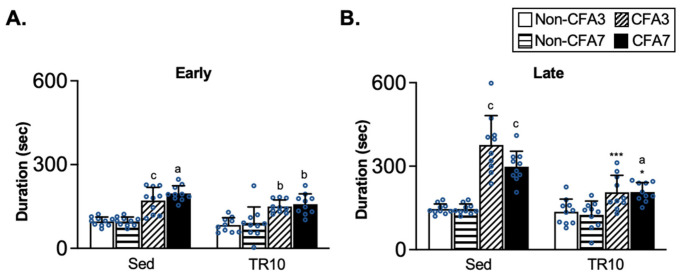
Effect of TR preconditioning on craniofacial pain-like behavior. Early phase (**A**) and late phase (**B**) in non-CFA and CFA mice under sedentary or TR10 conditions. *, *p* < 0.05; ***, *p* < 0.0001 versus the sedentary group within the corresponding treatment; a, *p* < 0.05; b, *p* < 0.001; c, *p* < 0.0001 versus the corresponding non-CFA control within the same treatment group. Open circles indicate individual data points.

#### 3.4.2. Late Phase (Figure 4B)

Post hoc analyses revealed that under sedentary conditions, the duration of craniofacial-pain-like behaviors was significantly greater in both the CFA3 and CFA7 groups than in the non-CFA3 and non-CFA7 groups (all groups, *p* < 0.0001). In contrast, within the TR10 group, craniofacial-pain-like behavioral durations were significantly greater in the CFA7 group (*p* < 0.039), but not in the CFA3 group (*p* < 0.17), than in the corresponding non-CFA groups. Of note, in the TR10 group, the duration of behaviors was significantly shorter than in the sedentary groups of CFA3 (*p* < 0.0001) and CFA7 (*p* < 0.01).

### 3.5. Effects of TR Preconditioning on Immunoreactivities for Various Markers

We quantified the number of immunopositive cells in the targeted brain regions on both the ipsilateral (left) and contralateral (right) sides relative to the CFA injection site. Representative microphotographs of immunoreactivities for histone H3 acetylation, HDAC1, and HDAC2 within distinct brain regions are shown in the corresponding sections below , while pCREB, FosB, and c-Fos are shown in Supplemental Figures S1–S4. The immunoreactivities were predominantly localized within cell nuclei, which is aligned with our earlier findings [10,15]. The region of interest is delineated, and the boxed areas indicate the sites analyzed quantitatively.

Statistical results for the between-subject factor (group comparisons) and within-subject factor (ipsilateral vs. contralateral side) are summarized in Supplemental Tables S3 and S4. Detailed effects of CFA-induced craniofacial inflammation under sedentary conditions are summarized in Supplementary Figure S5 and described in Supplementary Results (Text). Therefore, the following sections focus mainly on the effects of TR preconditioning under CFA-induced craniofacial inflammatory conditions.

#### 3.5.1. Amygdala

**Figure 5:**
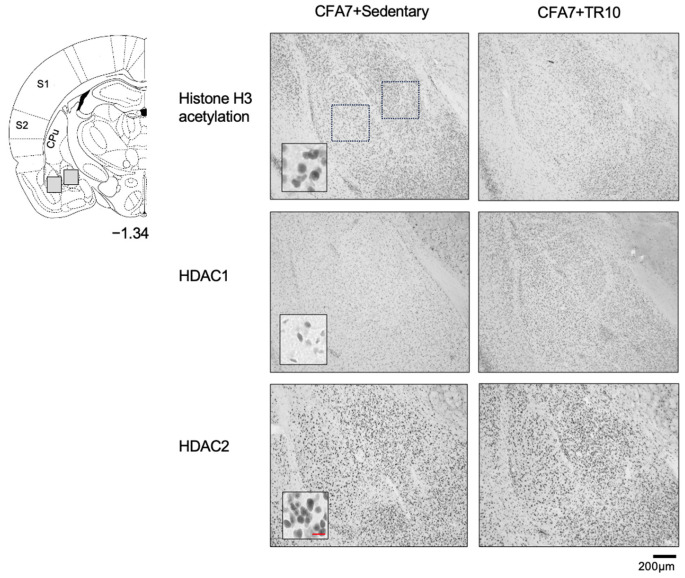
Microphotographs showing representative immunohistochemical images of histone H3 acetylation, HDAC1 expression, and HDAC2 expression in the BLA and CeA in CFA-treated mice under sedentary or TR10 conditions on Day +7. Cell counts for all markers were quantified within the same areas delineated by the dashed boxes in the histone H3 acetylation image. The insets feature a scale bar indicating a distance of 10 μm.

Figure 5 shows representative microphotographs of immunoreactivities for histone H3 acetylation, HDAC1, and HDAC2 specifically in two amygdala subregions (BLA and CeA).

- Histone H3 acetylation

**Figure 6:**
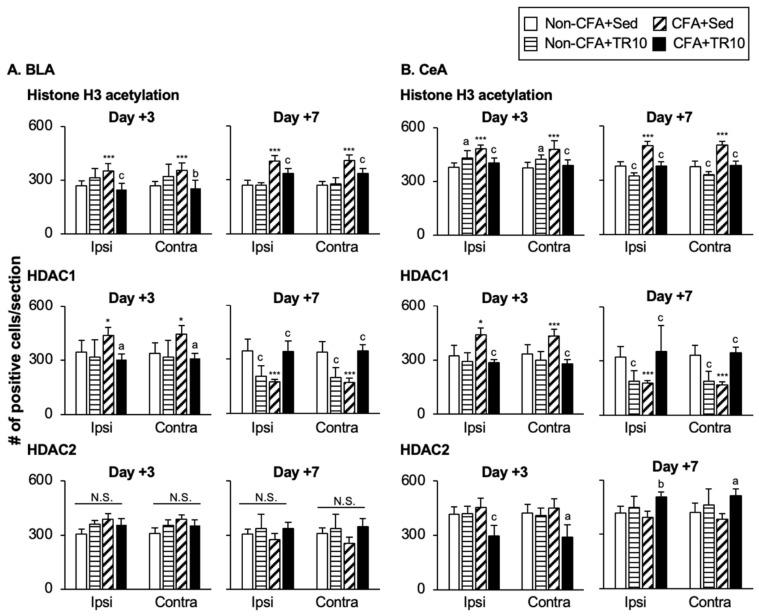
Effects of TR preconditioning on markers for epigenetic change in the amygdala. Quantification of histone H3 acetylation-, HDAC1-, and HDAC2-positive cells in the ipsilateral and contralateral BLA (**A**) and CeA (**B**) on Day +3 and Day +7 in non-CFA and CFA mice under sedentary or TR10 conditions. *, *p* < 0.05; ***, *p* < 0.0001 versus the corresponding non-CFA group; a, *p* < 0.05; b, *p* < 0.001; c, *p* < 0.0001 versus the sedentary group within the corresponding treatment. N.S., not significant.

In the CFA3 and CFA7 groups, the number of histone H3 acetylation-positive cells in both the BLA (Figure 6A) and CeA (Figure 6B) on both sides was significantly lower in the TR10 groups than in the corresponding sedentary groups.

In the non-CFA3 and non-CFA7 groups, the number of histone H3 acetylation-positive cells in the BLA was comparable between the TR10 and corresponding sedentary groups (*p* > 0.05). However, this pattern was not observed in the CeA, where the changes in histone H3 acetylation appeared to differ depending on the time point (Figure 6B). Specifically, the number of histone H3 acetylation-positive cells in the CeA was significantly higher in the TR10 group than in the sedentary group on Day 3, whereas it was significantly lower on Day 7.

b. HDAC1

The effect of TR10 differed between the CFA3 and CFA7 groups. In the CFA3 group, the number of HDAC1-positive cells in the BLA (*p* < 0.05, Figure 6A) and CeA (*p* < 0.0001, Figure 6B) was significantly lower in the TR10 group than in the sedentary group. In contrast, in the CFA7 group, the number of HDAC1-positive cells bilaterally in the BLA (*p* < 0.0001, Figure 6A) and CeA (*p* < 0.0001, Figure 6B) was significantly higher in the TR10 group than in the sedentary group.

In the non-CFA7 group, but not in the non-CFA3 group, the number of HDAC1-positive cells in the TR10 group was significantly lower in both the BLA and CeA than in the corresponding sedentary group (*p* < 0.0001).

c. HDAC2

In the CeA, the effects of TR10 differed between the CFA3 and CFA7 groups, as indicated by a significant decrease (CFA3 group) and increase (CFA7 group) in the number of HDAC2-positive cells (Figure 6B).

d. pCREB

**Figure 7:**
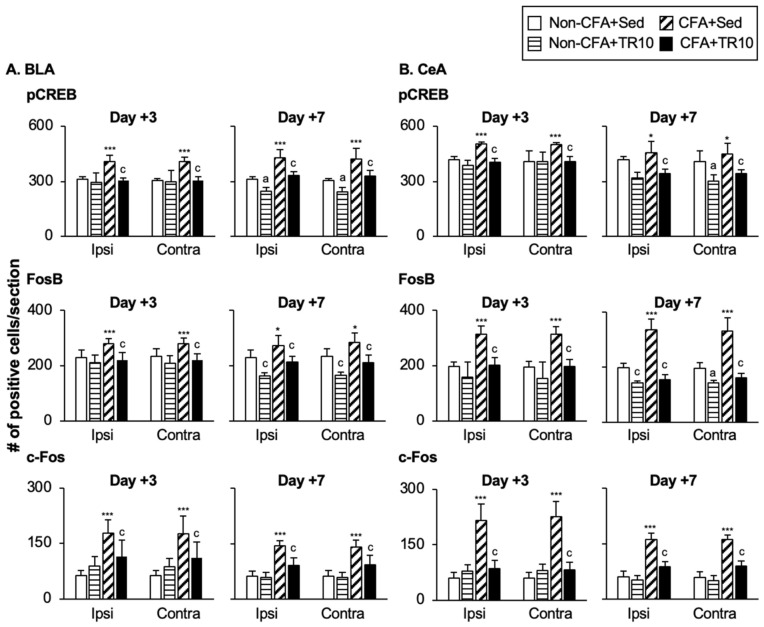
Effects of TR preconditioning on markers for neural activity in the amygdala. Quantification of the number of pCREB-, FosB-, and c-Fos-positive cells in the ipsilateral and contralateral BLA (**A**) and CeA (**B**) on Day +3 and Day +7 in non-CFA and CFA mice under sedentary or TR10 conditions. *, *p* < 0.05; ***, *p* < 0.0001 versus the corresponding non-CFA group; a, *p* < 0.05; c, *p* < 0.0001 versus the sedentary group within the corresponding treatment.

Under the CFA3 and CFA7 conditions, the number of pCREB-positive cells in both BLA and CeA of the TR10 group was significantly lower than that in the corresponding sedentary groups (both sides, *p* < 0.0001) (Figure 7A,B). In the non-CFA7 group, but not in the non-CFA3 group, the number of pCREB-positive cells in the BLA (both sides, *p* < 0.05) and the CeA (contralateral side, *p* < 0.05) of the TR10 group was significantly lower than that in the corresponding sedentary group.

e. FosB

Under the CFA3 and CFA7 conditions, the number of FosB-positive cells in both BLA and CeA of the TR10 group was significantly lower than that in the corresponding sedentary groups (both sides, *p* < 0.0001) (Figure 7A,B). In the non-CFA7 group, but not in the non-CFA3 group, the number of FosB-positive cells in the BLA (both sides, *p* < 0.0001) and the CeA (*p* < 0.00001 for the ipsilateral side, *p* < 0.05 for the contralateral side) of the TR10 group was significantly lower than that in the corresponding sedentary group.

f. c-Fos

Under the CFA3 and CFA7 conditions, the number of c-Fos-positive cells in both BLA and CeA of the TR10 group was significantly lower than that in the corresponding sedentary groups (both sides, *p* < 0.0001) (Figure 7A,B).

#### 3.5.2. Insular Cortex

**Figure 8:**
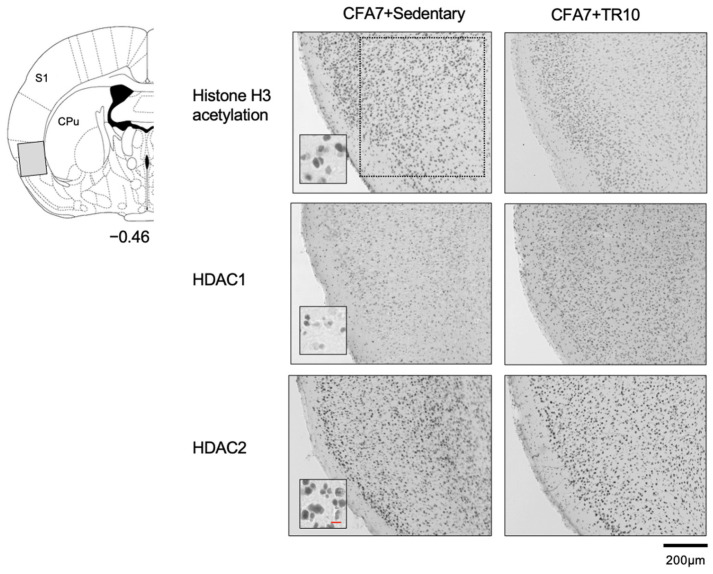
Microphotographs showing representative immunohistochemical images of histone H3 acetylation, HDAC1 expression, and HDAC2 expression within the insular cortex in CFA-treated mice under sedentary or TR10 conditions on Day +7. Cell counts for all markers were quantified within the same areas delineated by the dashed box shown in the histone H3 acetylation image. The insets feature a scale bar indicating a distance of 10 μm.

Figure 8 shows representative microphotographs of immunoreactivities for histone H3 acetylation, HDAC1, and HDAC2 in the insular cortex.

- Histone H3 acetylation

Under the CFA3 and CFA7 conditions, the number of histone H3 acetylation-positive cells in the IC of the TR10 group was significantly lower than that in the corresponding sedentary groups, except for that on the ipsilateral side of the CFA3 group. In the non-CFA7 group, the number of positive cells on the contralateral side of the TR10 group was significantly higher than that in the corresponding sedentary group (*p* < 0.05).

b. HDAC1

**Figure 9:**
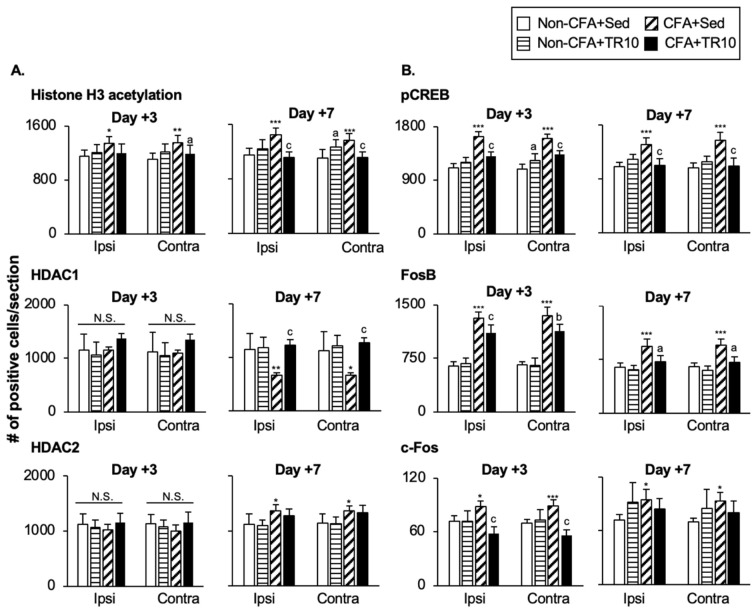
Effects of TR preconditioning on markers for epigenetic change and neural activity in the insular cortex. Quantification of the number of (**A**) histone H3 acetylation-, HDAC1-, and HDAC2- and (**B**) pCREB-, FosB-, and c-Fos-positive cells on the ipsilateral and contralateral sides on Day +3 and Day +7 in non-CFA and CFA mice under sedentary or TR10 conditions. *, *p* < 0.05; **, *p* < 0.001; ***, *p* < 0.0001 versus the corresponding non-CFA group; a, *p* < 0.05; b, *p* < 0.001; c, *p* < 0.0001 versus the sedentary group within the corresponding treatment. N.S., not significant.

In the CFA7 group, the number of HDAC1-positive cells was significantly higher in the TR10 group than in the sedentary group on both the ipsilateral and contralateral sides (all, *p* < 0.0001) (Figure 9A).

c. HDAC2

TR10 showed no significant effect on HDAC2 expression under both CFA and non-CFA conditions (Figure 9A).

d. pCREB

In the CFA3 and CFA7 groups, the number of pCREB-positive cells was significantly lower in the TR10 group than in the sedentary group on both the ipsilateral and contralateral sides (all, *p* < 0.0001) (Figure 9B). In the non-CFA3 group, the number of pCREB-positive cells on the contralateral side was significantly higher in the TR10 group than in the sedentary group (*p* < 0.05).

e. FosB

In the CFA3 and CFA7 groups, the number of FosB-positive cells was significantly lower in the TR10 group than in the sedentary group on both the ipsilateral and contralateral sides (Figure 9B).

f. c-Fos

In the CFA3 group, but not in the CFA7 group, the number of c-Fos-positive cells was significantly lower in the TR10 group than in the sedentary group on both the ipsilateral and contralateral sides (all, *p* < 0.0001) (Figure 9B).

#### 3.5.3. Hippocampal CA1

**Figure 10:**
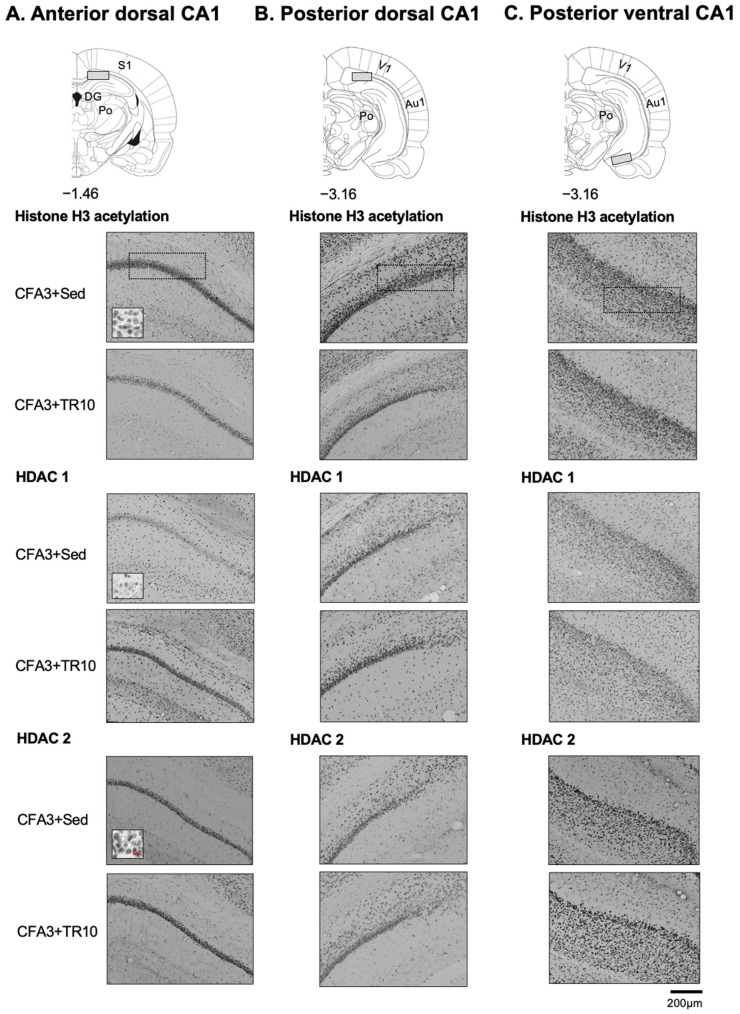
Microphotographs showing representative immunohistochemical images of histone H3 acetylation, HDAC1 expression, and HDAC2 expression within the hippocampal CA1 subregions: anterior dorsal CA1 (**A**), posterior dorsal CA1 (**B**), and posterior ventral CA1 (**C**) in CFA-treated mice under sedentary or TR10 conditions on Day +3. Cell counts for all markers were quantified within the same areas delineated within the dashed boxes shown in the histone H3 acetylation images. The insets feature a scale bar indicating a distance of 10 μm.

Figure 10 shows representative microphotographs of immunoreactivities for histone H3 acetylation, HDAC1, and HDAC2 in three subregions of the hippocampal CA1 (anterior dorsal, posterior dorsal, and ventral CA1).

**Figure 11:**
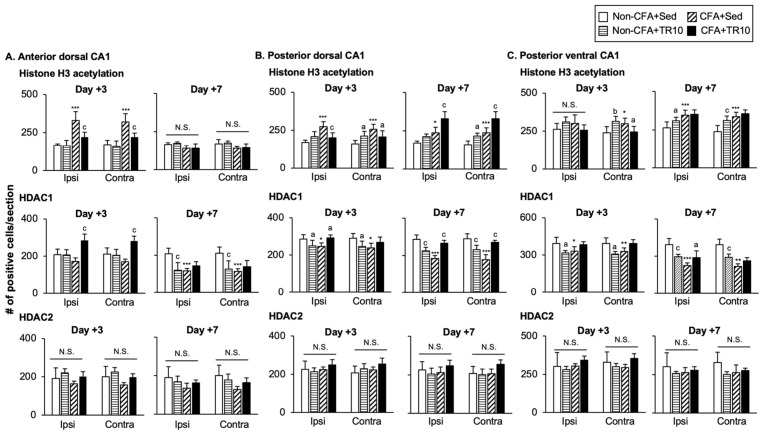
Effects of TR preconditioning on markers for epigenetic change in the hippocampus CA1. Quantification of the number of histone H3 acetylation-, HDAC1-, and HDAC2-positive cells in the ipsilateral and contralateral (**A**) anterior dorsal, (**B**) posterior dorsal, and (**C**) posterior ventral CA1 on Day +3 and Day +7 in non-CFA and CFA mice under sedentary or TR10 conditions. *, *p* < 0.05; **, *p* < 0.001; ***, *p* < 0.0001 versus the corresponding non-CFA group; a, *p* < 0.05; b, *p* < 0.001; c, *p* < 0.0001 versus the sedentary group within the corresponding treatment. N.S., not significant.

- Histone H3 acetylation

1. Anterior dorsal CA1 (Figure 11A)

In the CFA3 group, but not in the CFA7 group, the number of positive cells was significantly lower in the TR10 group than in the sedentary group on both the ipsilateral and contralateral sides.

2. Posterior dorsal CA1 (Figure 11B)

In the CFA3 group, the number of positive cells on both the ipsilateral and contralateral sides was significantly lower in the TR10 group than in the corresponding sedentary group (*p* < 0.0001 for the ipsilateral side, *p* < 0.05 for the contralateral side). In contrast, in the CFA7 group, the number of positive cells on both sides was significantly higher in the TR10 group than in the corresponding sedentary group (both sides, *p* < 0.0001). In the non-CFA3 and non-CFA7 groups, the number of positive cells on the contralateral, but not the ipsilateral, side was significantly higher in the TR10 group than in the corresponding sedentary group (*p* < 0.05).

3. Posterior ventral CA1 (Figure 11C)

In the CFA3 group, the number of positive cells on the contralateral side was significantly lower in the TR10 group than in the corresponding sedentary group (*p* < 0.05). In contrast, in the CFA7 group, the number of positive cells on both sides did not differ significantly between the TR10 and sedentary groups. In the non-CFA3 group, the number of positive cells on the contralateral side was significantly higher in the TR10 group than in the corresponding sedentary group (*p* < 0.001). Similarly, in the non-CFA7 group, the number of positive cells on the ipsilateral (*p* < 0.05) and contralateral (*p* < 0.0001) sides was significantly higher in the TR10 group than in the corresponding sedentary group.

b. HDAC1

1. Anterior dorsal CA1 (Figure 11A)

The number of positive cells in the CFA3 group was comparable to that in the non-CFA3 group. In the CFA3 group, the number of positive cells on both sides in the TR10 group was significantly higher than that in the corresponding sedentary group (*p* < 0.0001). In the CFA7 group, TR10 had no effect on HDAC1 expression. In the non-CFA7 group, the number of positive cells on both sides was significantly lower than that in the corresponding sedentary group (*p* < 0.0001).

2. Posterior dorsal CA1 (Figure 11B)

In the CFA3 group, the number of positive cells on the ipsilateral (*p* < 0.05) but not the contralateral side in the TR10 group was significantly higher than that in the corresponding sedentary group. In the CFA7 group, the number of positive cells on both sides in the TR10 group (*p* < 0.0001) was significantly higher than that in the corresponding sedentary group. In the non-CFA3 (*p* < 0.05) and non-CFA7 (*p* < 0.0001) groups, the number of positive cells on both sides in the TR10 group was significantly lower than that in the corresponding sedentary group.

3. Posterior ventral CA1 (Figure 11C)

In the CFA3 group, TR10 displayed no significant effect. In the CFA7 group, the number of positive cells on the ipsilateral but not the contralateral side in the TR10 group was significantly higher than that in the corresponding sedentary group (*p* < 0.05). In the non-CFA3 and non-CFA7 groups, the number of positive cells on both sides in the TR10 group was significantly lower than in the corresponding sedentary group.

c. HDAC2

The number of HDAC2-positive cells in the three CA1 subregions was not significantly affected by TR10 in any treatment group (Figure 11).

d. pCREB

**Figure 12:**
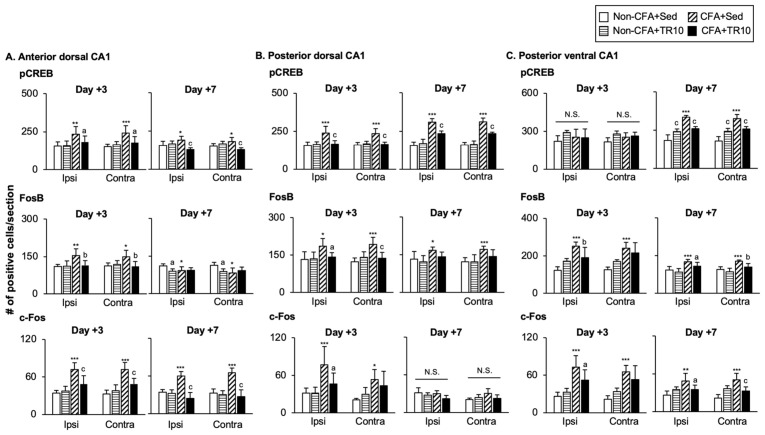
Effects of TR preconditioning on markers for neural activity in the hippocampus CA1. Quantification of the number of pCREB-, FosB-, and c-Fos-positive cells in the ipsilateral and contralateral (**A**) anterior dorsal, (**B**) posterior dorsal, and (**C**) posterior ventral CA1 on Day +3 and Day +7 in non-CFA and CFA mice under sedentary or TR10 conditions. *, *p* < 0.05; **, *p* < 0.001; ***, *p* < 0.0001 versus the corresponding non-CFA group; a, *p* < 0.05; b, *p* < 0.001; c, *p* < 0.0001 versus the sedentary group within the corresponding treatment. N.S., not significant.

1. Anterior dorsal CA1 (Figure 12A)

In both the CFA3 (both sides, *p* < 0.05) and CFA7 (both sides, *p* < 0.0001) groups, the number of positive cells in the TR10 group was significantly lower than that in the corresponding sedentary group.

2. Posterior dorsal CA1 (Figure 12B)

In both the CFA3 (both sides, *p* < 0.0001) and CFA7 (both sides, *p* < 0.0001) groups, the number of positive cells in the TR10 group was significantly lower than that in the corresponding sedentary group.

3. Posterior ventral CA1 (Figure 12C)

In the CFA7 group, the number of positive cells in the TR10 group was significantly lower than that in the corresponding sedentary group (both sides, *p* < 0.0001). In the non-CFA7 group, the number of positive cells was significantly higher than that in the corresponding sedentary group (both sides, *p* < 0.0001).

e. FosB

1. Anterior dorsal CA1 (Figure 12A)

In the CFA3 group, the number of positive cells in the TR10 group was significantly lower than that in the corresponding sedentary group (both sides, *p* < 0.001); however, it was not altered in the CFA7 group. In the non-CFA7 group, but not in the non-CFA3 group, the number of positive cells in the TR10 group was significantly lower than that in the corresponding sedentary group (both sides, *p* < 0.05).

2. Posterior dorsal CA1 (Figure 12B)

In the CFA3 group, the number of positive cells in the TR10 group was significantly lower than that in the corresponding sedentary group, while in the CFA7 group, it was not significantly altered compared with the corresponding sedentary group.

3. Posterior ventral CA1 (Figure 12C)

In the CFA3 group, the number of positive cells on the ipsilateral side (*p* < 0.001) in the TR10 group was significantly lower than that in the corresponding sedentary group. In the CFA7 group, the number of positive cells on the ipsilateral (*p* < 0.05) and contralateral (*p* < 0.001) sides in the TR10 group was significantly lower than that in the corresponding sedentary group.

f. c-Fos

1. Anterior dorsal CA1 (Figure 12A)

In the CFA3 and CFA7 groups, the number of positive cells on both sides in the TR10 group was significantly lower than that in the corresponding sedentary group (all, *p* < 0.0001).

2. Posterior dorsal CA1 (Figure 12B)

In the CFA3 group, the number of positive cells on the ipsilateral side in the TR10 group was significantly lower than that in the corresponding sedentary group (*p* < 0.05). In the CFA7 group, no significant effect of CFA or TR10 was observed.

3. Posterior ventral CA1 (Figure 12C)

In the CFA3 group, the number of positive cells on the ipsilateral side (*p* < 0.05) in the TR10 group was significantly lower than that in the corresponding sedentary group, while that in the CFA7 group on both the ipsilateral (*p* < 0.05) and contralateral (*p* < 0.0001) sides was significantly lower than that in the corresponding sedentary CFA7 group.

#### 3.5.4. Primary Motor Cortex

**Figure 13:**
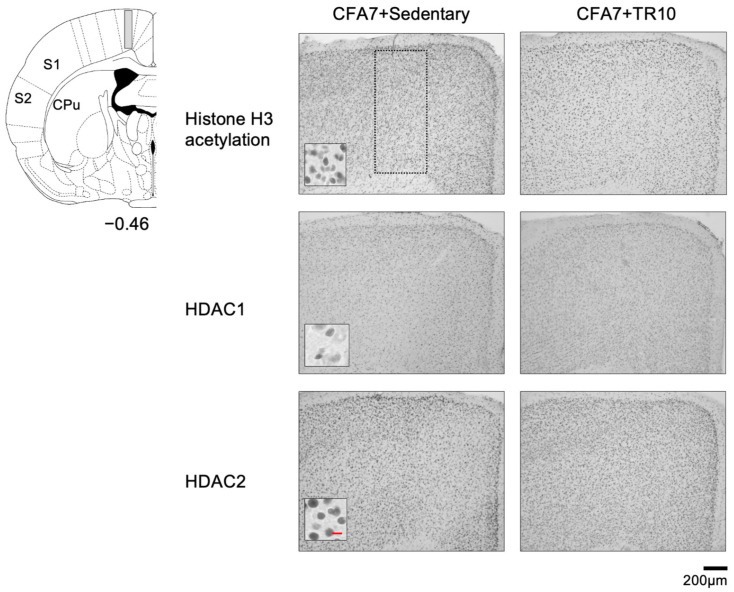
Microphotographs showing representative immunohistochemical images of histone H3 acetylation, HDAC1 expression, and HDAC2 expression within the M1 in CFA-treated mice under sedentary or TR10 conditions on Day +7. Cell counts for all markers were quantified within the same areas delineated by the dashed box shown in the histone H3 acetylation image. The insets feature a scale bar indicating a distance of 10 μm.

Figure 13 shows representative microphotographs of immunoreactivities for histone H3 acetylation, HDAC1, and HDAC2 in the M1.

- Histone H3 acetylation

**Figure 14:**
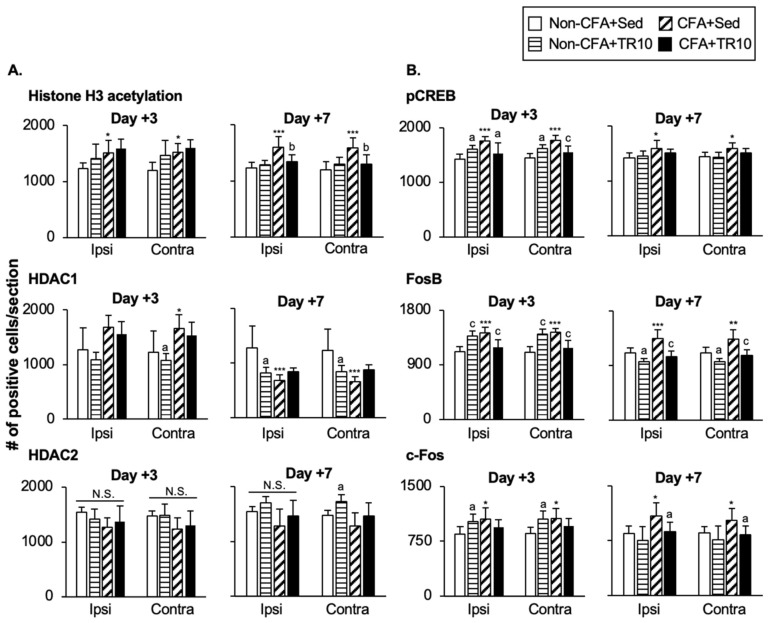
Effects of TR preconditioning on markers for epigenetic change and neural activity in the M1. Quantification of the number of (**A**) histone H3 acetylation-, HDAC1-, and HDAC2- and (**B**) pCREB-, FosB-, and c-Fos-positive cells on the ipsilateral and contralateral sides on Day +3 and Day +7 in non-CFA and CFA mice under sedentary or TR10 conditions. *, *p* < 0.05; **, *p* < 0.001; ***, *p* < 0.0001 versus the corresponding non-CFA group; a, *p* < 0.05; b, *p* < 0.001; c, *p* < 0.0001 versus the sedentary group within the corresponding treatment. N.S., not significant.

In the CFA7 group, the number of positive cells on both the ipsilateral and contralateral sides was significantly lower in the TR10 group than in the corresponding sedentary group (both sides, *p* < 0.001; Figure 14A). In contrast, in the CFA3 group, TR10 showed no significant effect on histone H3 acetylation expression.

b. HDAC1

TR10 showed no significant effect on HDAC1 expression in the CFA3 and CFA7 groups. In the non-CFA3 and non-CFA7 groups, the number of positive cells was significantly lower in the TR10 group than in the corresponding sedentary group, except for that on the ipsilateral side in the non-CFA3 group.

c. HDAC2

TR10 showed no significant effect on HDAC2 expression in the CFA3 or CFA7 group. In the non-CFA7 group, but not in the non-CFA3 group, the number of positive cells on the contralateral side was significantly higher in the TR10 group than in the corresponding sedentary group (*p* < 0.05; Figure 14A).

d. pCREB

In the CFA3 group, the number of positive cells on both the ipsilateral and contralateral sides was significantly lower in the TR10 group than in the corresponding sedentary group. In contrast, TR10 showed no significant effect on pCREB expression in the CFA7 group (Figure 14B). In the non-CFA3 group, the number of pCREB-positive cells on both sides was significantly higher in the TR10 group than in the corresponding sedentary group (*p* < 0.05).

e. FosB

In the CFA3 and CFA7 groups, the number of positive cells on both the ipsilateral and contralateral sides was significantly lower in the TR10 group than in the corresponding sedentary group (all, *p* < 0.0001; Figure 14B). In the non-CFA3 group, the number of FosB-positive cells on both sides was significantly higher in the TR10 group than in the corresponding sedentary group (*p* < 0.0001). In the non-CFA7 group, the number of positive cells on both sides was significantly lower in the TR10 group than in the corresponding sedentary group (*p* < 0.05).

f. c-Fos

In the CFA7 group, the number of c-Fos-positive cells on both the ipsilateral and contralateral sides was significantly lower in the TR10 group than in the corresponding sedentary group (both sides, *p* < 0.05; Figure 14B). In contrast, TR10 showed no significant effect on c-Fos expression in the CFA3 group. In the non-CFA3 group, the number of c-Fos-positive cells on both sides was significantly higher in the TR10 group than in the corresponding sedentary group (*p* < 0.05).

### 3.6. Correlation Analysis

As presented in Table 1, correlation analyses were conducted separately within each treatment condition detailed below.

#### 3.6.1. Non-CFA Groups

In almost all subregions, no significant correlations were identified. However, significant correlations with pain-like behaviors were observed between c-Fos levels in the M1 region of the non-CFA3 group and between FosB levels in the posterior ventral CA1 region of the non-CFA3 and non-CFA7 groups.

#### 3.6.2. CFA Groups

In all subregions of the amygdala and insular cortex, significant correlations were observed in both the CFA3 and CFA7 groups, except for c-Fos in the insular cortex of the CFA7 group.

In the hippocampal CA1 region, the results appeared to be more variable and dependent on the marker and subregions. In the anterior dorsal CA1, histone H3 acetylation and c-Fos expression were correlated with pain-like behaviors in the CFA3 group, whereas pCREB and c-Fos levels were correlated with pain-like behaviors in the CFA7 group. In the posterior dorsal CA1, all markers (except c-Fos) for CFA3 and CFA7 groups were correlated with behaviors. In the posterior ventral CA1, no significant correlations were identified in the CFA3 group, whereas in the CFA7 group, pCREB and FosB levels were significantly correlated with pain-like behaviors.

In the M1, the levels of pCREB and FosB in the CFA3 group, and that of FosB in the CFA7 group, were significantly correlated.

**Figure 15:**
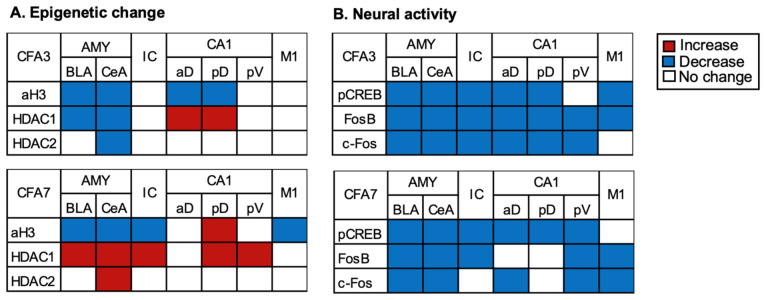
Summary diagram illustrating the effects of TR preconditioning on epigenetic- and neural-activity-related markers under CFA conditions. This schematic summarizes significant ipsilateral changes in marker expression induced by TR10 on Day 3 (CFA3) and Day 7 (CFA7), expressed relative to the corresponding CFA–sedentary groups. Red and blue shading indicate significant increases and decreases, respectively, whereas blank boxes indicate no significant change compared with the corresponding CFA–sedentary groups. Abbreviations: AMY, amygdala; BLA, basolateral amygdala; CeA, central amygdala; IC, insular cortex; aD, anterior dorsal CA1; pD, posterior dorsal CA1; pV, posterior ventral CA1; M1, primary motor cortex; CFA, complete Freund’s adjuvant; TR10, treadmill run preconditioning.

Finally, to provide an integrated overview, Figure 15 illustrates the direction of statistically significant changes induced by TR preconditioning on epigenetic and neural activity markers across brain regions and time points under CFA conditions.

## 4. Discussion

The present study addressed two main objectives using a persistent craniofacial pain model. First, we examined whether repeated daily TR performed before the onset of craniofacial inflammation could exert preventive effects on craniofacial-pain- and anxiety-like behaviors. Second, we investigated which brain regions might exhibit prolonged changes in brain responses using multiple markers for epigenetic changes and neural activities even after the cessation of daily TR conditioning.

### 4.1. Effects of TR Preconditioning on Behavioral Responses

Our present study demonstrated that even three and seven days after the cessation of TR, anxiety-like behaviors were reduced in the CFA3 group and, in several measures, remained suppressed in the CFA7 group. Our findings are consistent with previous reports demonstrating that the anxiolytic effects induced by physical exercise persist beyond the exercise period in both rodents and humans [44,45]. Similarly, our TR preconditioning paradigm attenuated craniofacial-pain-like behaviors, with the most robust effects observed during the late phase of the formalin response. Such selective suppression of late-phase formalin-evoked craniofacialpain-like behaviors by TR has also been reported in mice subjected to repeated social defeat stress [9]. Therefore, the reduction in late-phase responses supports the idea that TR preconditioning modulates the neural basis for nociception in the central rather than peripheral nervous system [34,46]. Importantly, the present study provides novel evidence that such prolonged effects on craniofacial-pain-like behaviors, along with anxiety-like behaviors, can also occur under CFA-induced persistent craniofacial inflammation. Although motor performance was not directly assessed, the possibility that CFA-induced locomotor impairment substantially influenced the behavioral outcomes appears limited, because TR preconditioning attenuated several anxiety- (e.g., increased motor activity in the open-field test) and pain-like (decreased grooming motor activity) behavioral responses three and seven days after exercise cessation.

### 4.2. Effects of TR Preconditioning on Brain Responses

Evidence indicates that physical exercise exerts inhibitory effects on both pain- and anxiety-related brain responses. In most previous studies, exercise interventions were initiated after inflammation [3,4] or nerve injury [47] and continued on a daily basis. Consequently, the impact of physical exercise performed prior to pain-related neural responses that emerge after peripheral inflammation is not well-known.

Our findings provide novel evidence that TR preconditioning produces sustained, region-specific modulation of marker levels for epigenetic changes and neural activity in the amygdala, insular cortex, CA1 hippocampus, and M1, such that these modulatory effects remain detectable under CFA-induced craniofacial inflammation using a well-known craniofacial pain model. Although TR preconditioning might not directly reflect most clinical situations in patients with already established craniofacial pain, preventive exercise conditioning is increasingly recognized as an important strategy to enhance physical resilience before predictable physiological stress, such as surgery or injury [48,49].

In this study, TR preconditioning produced region- and time-dependent effects on histone H3 acetylation. The present results do not explain the neural basis underlying these region-specific temporal patterns. Nevertheless, several possibilities might be considered. The amygdala is critically involved in the relatively stable encoding of emotional salience and threat-related memories [50,51], which might permit TR-induced epigenetic changes to persist across both CFA3 and CFA7 time points. In contrast, the insular cortex, a region implicated in the affective and interoceptive dimensions of persistent pain [52], might require a more prolonged inflammatory state before the modulatory effects of TR preconditioning become detectable.

In the hippocampal CA1, the effects of TR preconditioning appeared more transient and subregion-dependent. The suppressive effects observed in the anterior and posterior dorsal CA1 in CFA3 were not maintained in CFA7, suggesting that TR-induced modulation in CA1 may be less stable than that in the amygdala. This observation might be related to the fact that the hippocampal CA1 region exhibits context- and time-dependent neural changes that are inherently more dynamic [53,54,55]. Importantly, increased marker expression does not necessarily indicate abnormal neural activation, because physical exercise itself can induce neural change [56,57]. Indeed, under non-CFA conditions, TR preconditioning altered histone H3 acetylation in several regions without producing detectable pain- or anxiety-like behavioral changes. This suggests that TR-induced epigenetic modulation in the CA1 region might reflect physiological neural change rather than a direct pathological or anti-nociceptive response.

Additionally, in the M1 region, the effects of TR preconditioning on histone H3 acetylation were time-dependent, with inhibitory effects observed in the CFA7 group but not in the CFA3 group. Although the basis for this delayed TR effect remains unclear, these findings suggest that TR preconditioning might induce modulatory changes in the M1 beyond craniofacial motor representations.

Reductions in histone acetylation are generally associated with increased HDAC activity [58]. Consistent with this concept, the increased HDAC1 expression observed in some brain regions might partly explain the reduction in histone H3 acetylation after TR preconditioning. However, this relationship was not consistent across all regions and time points, suggesting that TR-induced epigenetic changes are unlikely to be driven by a single HDAC1-dependent mechanism. Other possible mechanisms include the involvement of other HDAC isoforms, such as HDAC5 and HDAC6 [59], as well as alterations in histone acetyltransferase activity [58]. Although these possibilities remain speculative, the observed changes in these markers suggest that TR preconditioning might exert modulatory effects on epigenetic regulation in the brain.

Besides epigenetic changes, we also determined the effects of TR preconditioning on the level of markers for neural activity. Previous reports supported the notion that physical exercise could alter the expression of pCREB, FosB, and c-Fos in the brain [60,61,62]. In the present study, TR preconditioning generally reduced these markers across several pain-related brain regions during CFA-induced inflammatory conditions, suggesting that prior exercise may suppress neural responses associated with persistent craniofacial inflammation.

Analyzing multiple neural-activity-related markers provides complementary information because they reflect distinct temporal and molecular aspects of neural responses. In our results, the inhibitory effects of TR were robust in the amygdala, while in the insular cortex, hippocampal CA1, and M1, they were time-dependent and region-specific. These results support the possibility that TR-induced reductions in neural marker expression might be functionally related to the attenuation of craniofacial-pain-like behaviors under conditions of craniofacial inflammation. Ample evidence supports that normalization of neural activity in the amygdala [63,64], insular cortex [12,65], hippocampal CA1 [47], and M1 [66] attenuates those behaviors in various pain models.

Our current findings of neural activity do not allow for any direct linkage with epigenetic changes because this is beyond the scope of our study. However, in contrast to the variable effects observed for epigenetic markers, TR preconditioning exerted more consistent effects on neural activity markers, reducing their levels across most brain regions under both CFA conditions.

### 4.3. Limitations

First, the present findings do not demonstrate a direct causal relationship between the behavioral outcomes and the immunohistochemical changes. The correlation analyses presented in Table 1 provide indirect evidence that several epigenetic- and neural-activity-related marker expression levels were correlated with craniofacial-pain-like behaviors in the CFA3 and CFA7 groups; therefore, these results should be interpreted as associative rather than causal. Future studies using selective pharmacological or genetic manipulation of these signaling pathways will be necessary to clarify their causal roles.

Second, our results do not identify the specific cell populations expressing these markers. For example, evidence revealed that both neurons and glial cells express histone H3 acetylation and HDAC1, of which levels were affected by TR [67]. Accordingly, the present study cannot determine the overall functional state, such as whether the observed changes reflect excitatory or inhibitory neural tone.

Third, our results do not demonstrate the basis responsible for the prolonged effects of TR on behavioral and brain responses. Previous studies suggest that sustained effects of physical exercise might involve changes in neural signaling, neurotrophic factors, brain metabolism, and epigenetic regulation [68,69,70]. Furthermore, as shown in Figure 1E, alterations in brain lactate levels are also observed, even 3 and 7 days after the cessation of a 10-day TR conditioning, which has been documented to regulate brain responses [41,42,43]. Although the functional roles of lactate in craniofacial nociception remain unclear, TR preconditioning exhibited a prolonged effect on lactate metabolism in the brain at least.

Fourth, this study employed only male mice, which represents an important limitation because craniofacial pain conditions are more prevalent in females [71], and sex-dependent differences have also been reported in stress-induced modulation of trigeminal nociceptive processing [72,73]. Therefore, the present findings might reflect the partial characteristics of human craniofacial pain conditions and should not be generalized to female mice [74]. Future studies including female mice are necessary to determine whether the preventive effects of TR preconditioning on craniofacial-pain-like behaviors and associated brain responses are sex-specific.

## 5. Conclusions

Repeated TR preconditioning exerted prolonged preventive effects on craniofacial-pain- and anxiety-like behaviors even several days after the cessation of exercise. These behavioral effects were accompanied by sustained alterations in epigenetic and neural activity markers in multiple pain-related brain regions, including the amygdala, insular cortex, hippocampal CA1, and M1. Together, these findings suggest that repeated TR induces persistent changes in brain responses that might contribute to resilience against subsequent craniofacial inflammatory pain.

## Figures and Tables

**Table 1:** Spearman’s correlation analysis between craniofacial-pain-like behaviors and histone H3 acetylation, pCREB, FosB, and c-Fos in the evaluated brain regions. The correlation between the time spent performing formalin-evoked grooming behaviors and the number of each immunopositive cell/section was assessed. Detailed sample sizes are presented in Supplemental Table S1C.

	Non-CFA3 (Sed: n = 6–7 TR10 n = 9–10)		Non-CFA7 (Sed: n = 6–7 TR10 n = 9–10)		CFA3 (Sed: n = 5–9 TR10 n = 7–10)		CFA7 (Sed: n = 10 TR10 n = 9)	
	r	*p*-Value	r	*p*-Value	r	*p*-Value	r	*p*-Value
Histone H3 acetylation								
Amygdala								
BLA	0.131	0.628	0.295	0.267	0.749	0.001 **	0.773	0.002 *
CeA	−0.594	0.150	0.220	0.413	0.630	0.012 *	0.731	0.0001 ***
Insular cortex								
	−0.233	0.386	0.047	0.862	0.642	0.010 *	0.717	0.001 **
Hippocampal CA1								
aD	0.208	0.441	−0.140	0.604	0.704	0.003 *	0.072	0.770
pD	−0.109	0.688	−0.236	0.379	0.715	0.003 *	−0.517	0.023 *
pV	−0.134	0.621	−0.090	0.740	0.507	0.060	−0.035	0.888
Primary motor cortex								
	−0.430	0.095	−0.280	0.293	−0.170	0.545	0.453	0.500
pCREB								
Amygdala								
BLA	0.037	0.889	0.295	0.251	0.764	0.0001 ***	0.644	0.003 *
CeA	0.026	0.304	−0.132	0.615	0.742	0.0001 ***	0.680	0.001 **
Insular cortex								
	−0.061	0.815	0.138	0.598	0.778	0.0001 ***	0.720	0.001 **
Hippocampal CA1								
aD	0.117	0.656	0.114	0.662	0.347	0.146	0.749	0.0001 ***
pD	0.174	0.503	−0.188	0.469	0.695	0.001 **	0.694	0.001 **
pV	−0.350	0.169	0.189	0.467	0.096	0.697	0.755	0.0001 ***
Primary motor cortex								
	−0.410	0.103	−0.170	0.515	0.778	0.003 *	−0.008	0.974
FosB								
Amygdala								
BLA	−0.003	0.991	0.138	0.598	0.588	0.010 **	0.656	0.002 *
CeA	0.324	0.221	0.048	0.855	0.753	0.0001 ***	0.681	0.001 **
Insular cortex								
	0.004	0.987	0.334	0.190	0.477	0.045 *	0.668	0.002 *
Hippocampal CA1								
aD	0.222	0.408	−0.123	0.638	0.452	0.060	0.017	0.945
pD	0.038	0.888	−0.133	0.611	0.710	0.001 **	0.462	0.046 *
pV	−0.650	0.006 *	−0.498	0.042 *	0.412	0.089	0.488	0.034 *
Primary motor cortex								
	−0.387	0.139	0.059	0.822	0.674	0.002 *	0.706	0.001 **
c-Fos								
Amygdala								
BLA	−0.261	0.347	0.293	0.289	0.851	0.0001 ***	0.659	0.002 *
CeA	−0.247	0.375	−0.052	0.854	0.876	0.0001 ***	0.734	0.0001 ***
Insular cortex								
	0.232	0.406	−0.146	0.605	0.783	0.014 *	0.091	0.713
Hippocampal CA1								
aD	−0.163	0.561	0.116	0.682	0.644	0.024 *	0.597	0.007 *
pD	0.322	0.242	0.190	0.497	0.546	0.006 *	0.280	0.245
pV	−0.402	0.137	−0.443	0.098	0.529	0.077	0.443	0.057
Primary motor cortex								
	−0.570	0.026 *	0.269	0.333	0.483	0.110	0.383	0.106

*, *p* < 0.05; **, *p* < 0.001; ***, *p* < 0.0001; Spearman’s correlation coefficient (r). Abbreviations: aD, anterior dorsal CA1; BLA, basolateral amygdala; CeA, central amygdala; pD, posterior dorsal CA1; pV, posterior ventral CA1; Sed, sedentary; TR10, treadmill run preconditioning.

## Data Availability

The original contributions presented in this study are included in the article/Supplementary Materials. Further inquiries can be directed to the corresponding author.

## Supplementary Materials

The following supporting information can be downloaded at https://www.mdpi.com/article/10.3390/biomedicines14071576/s1: Table S1: Details on number of animals used in the present study; Table S2: Key resources used in this study; Table S3: Statistical data for the main effects for between-subject factors (group comparisons) and within-subject factors (ipsilateral versus contralateral sides) in the CFA3 group; Table S4: Statistical data for the main effects for between-subject factors (group comparisons) and within-subject factors (ipsilateral versus contralateral sides) in the CFA7 group; Figure S1: Microphotographs displaying pCREB, FosB, and c-Fos expression within the basolateral amygdala (BLA) and central amygdala (CeA); Figure S2: Microphotographs displaying pCREB, FosB, and c-Fos expression in the insular cortex (IC); Figure S3: Microphotographs displaying pCREB, FosB, and c-Fos expression within the hippocampal CA1 region; Figure S4: Microphotographs displaying pCREB, FosB, and c-Fos expression in the primary motor cortex (M1); Figure S5: Summary diagram illustrating the effects of CFA-induced craniofacial inflammation on epigenetic changes and neural activity markers in the brain under sedentary conditions; Supplementary Results (Text): Detailed results of the effects of CFA-induced craniofacial inflammation under sedentary conditions.

## Abbreviations

The following abbreviations are used in this manuscript:

aD	Anterior dorsal CA1
aH3	Histone H3 acetylation
BLA	Basolateral subregion of amygdala
CA1	Cornu ammonis 1
CeA	Central subregion of amygdala
CFA	Complete Freund’s adjuvant
Contra	Contralateral side
EPM	Elevated plus maze
HDAC1	Histone deacetylase 1
HDAC2	Histone deacetylase 2
IC	Insular cortex
IHC	Immunohistochemistry
Ipsi	Ipsilateral side
M1	Primary motor cortex
OF	Open field
pCREB	Phosphorylated cAMP response element-binding protein
pD	Posterior dorsal CA1
pV	Posterior ventral CA1
TR	Treadmill run
TR10	Treadmill run preconditioning
SD	Standard deviation
Sed	Sedentary
