# Supplementary material for: Repeated Treadmill Run Preconditioning Induces Prolonged Attenuation of Craniofacial Pain-like Behaviors and Changes in Brain Responses Associated with Persistent Craniofacial Inflammation in Male Mice
Source: Biomedicines. 2026 Jul 14;14(7):1576. doi: 10.3390/biomedicines14071576 (PMC13407325; doi:10.3390/biomedicines14071576)
Supplement: Supplementary file 1 [file biomedicines-14-01576-s001.zip › SFigures 1-5R1 0708am9.pdf]

Supplemental Figures:

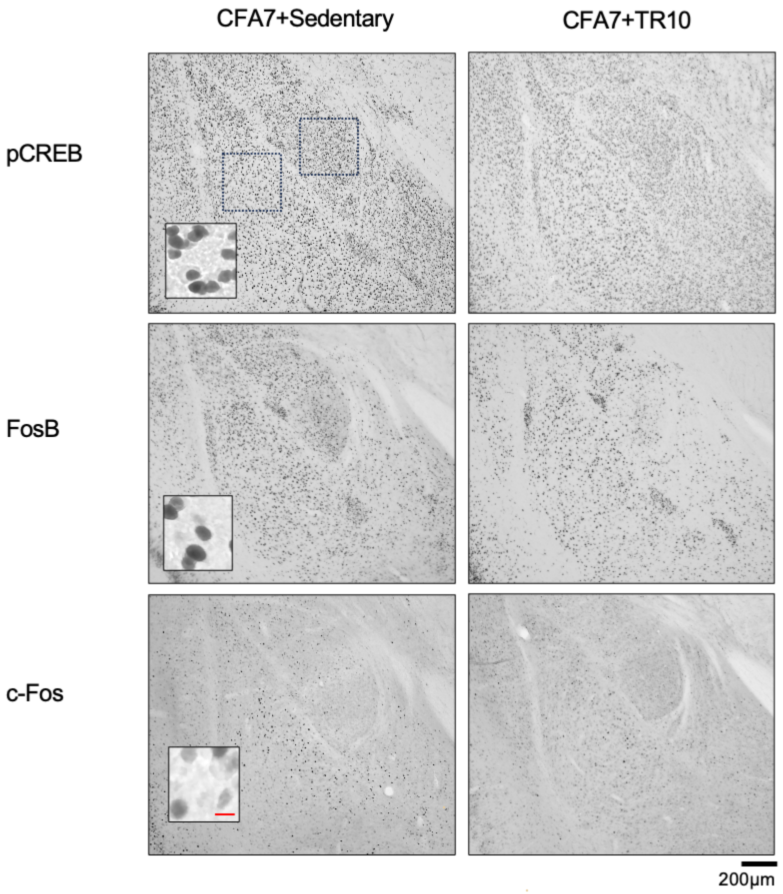

Figure S1. Microphotographs displaying pCREB, FosB, and c-Fos expression within the basolateral amygdala (BLA) and central amygdala (CeA). Representative immunohistochemical images of pCREB, FosB, and c-Fos expression in the amygdala (ipsilateral to CFA) in CFA-treated mice under sedentary or TR10 conditions at Day +7. These immunoreactivities were predominantly localized within cell nuclei, which are consistent with our previous reports. Cell counts for all markers were quantified within the same areas delineated by the boxes in the pCREB images. The scale bar in the insets represents 10 μm.

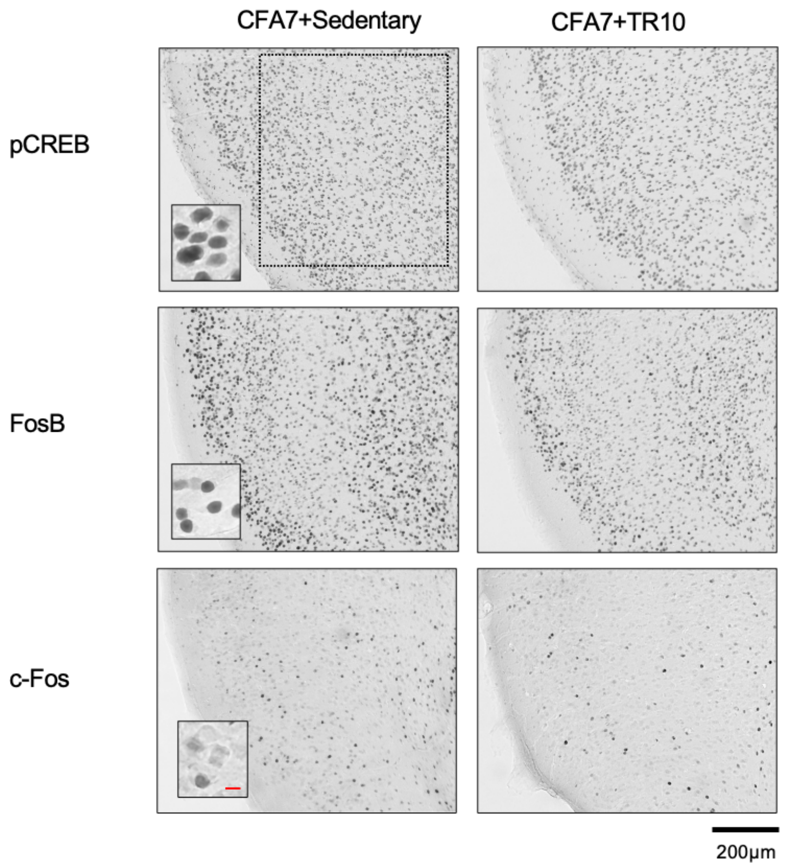

Figure S2. Microphotographs displaying pCREB, FosB, and c-Fos expression in the insular cortex (IC). Representative immunohistochemical images of pCREB, FosB, and c-Fos expression in the insular cortex (ipsilateral to CFA) in CFA-treated mice under sedentary or TR10 conditions at Day +7. These immunoreactivities were predominantly localized within cell nuclei. Cell counts for all markers were quantified within the same areas delineated by the boxes shown in the pCREB images. The scale bar in the insets represents 10 μm.

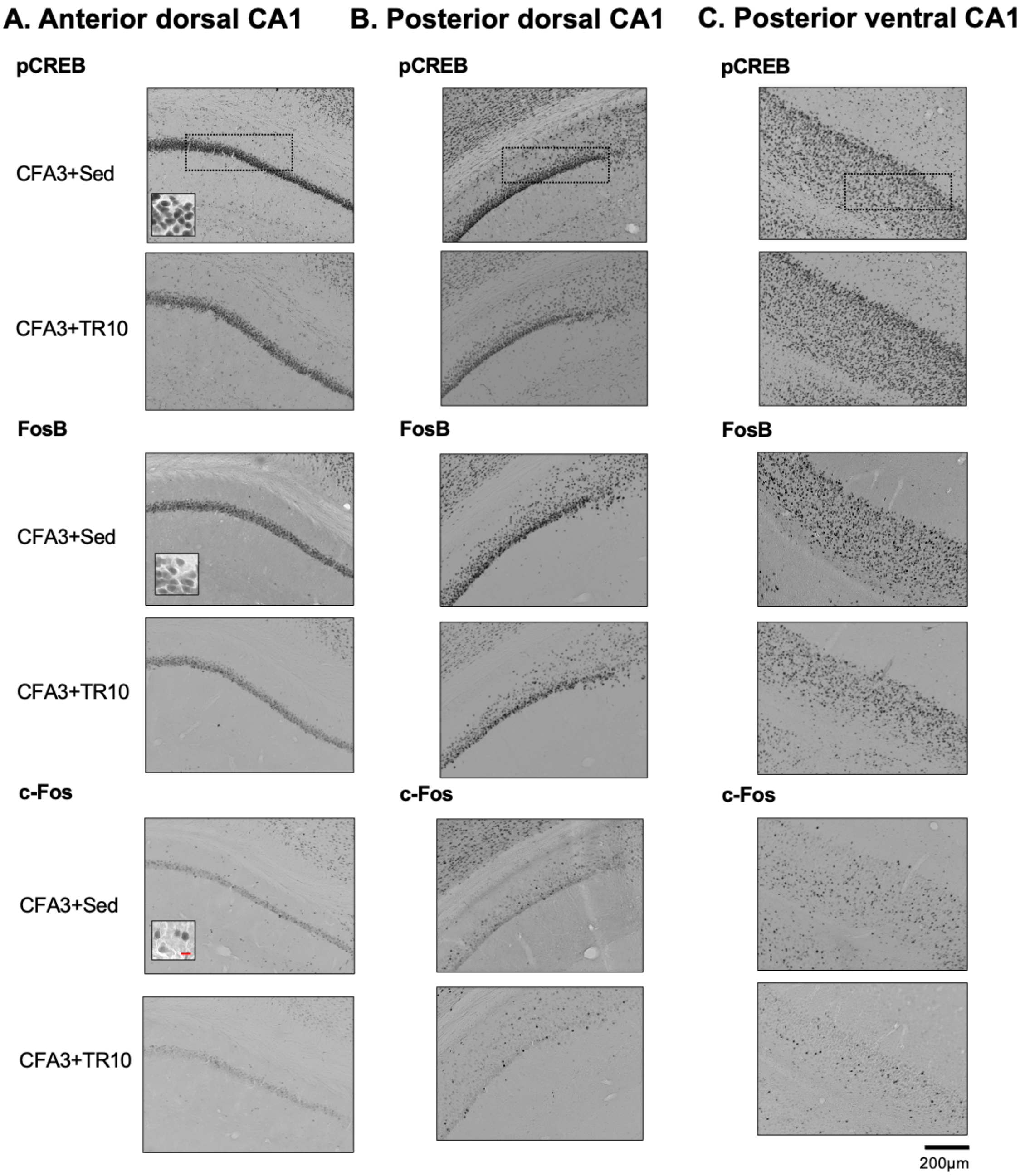

Figure S3. Microphotographs displaying pCREB, FosB, and c-Fos expression within the hippocampal CA1 region. representative immunohistochemical images of pCREB, FosB, and c-Fos expression in the anterior dorsal CA1 (**A**), posterior dorsal CA1 (**B**), and posterior ventral CA1 (**C**) in CFA-treated mice under sedentary or TR10 conditions at Day +3. These immunoreactivities were predominantly localized within cell nuclei. Cell counts for all markers were quantified within the same areas delineated by the boxes shown in the pCREB images. The scale bar in the insets represents 10 μm.

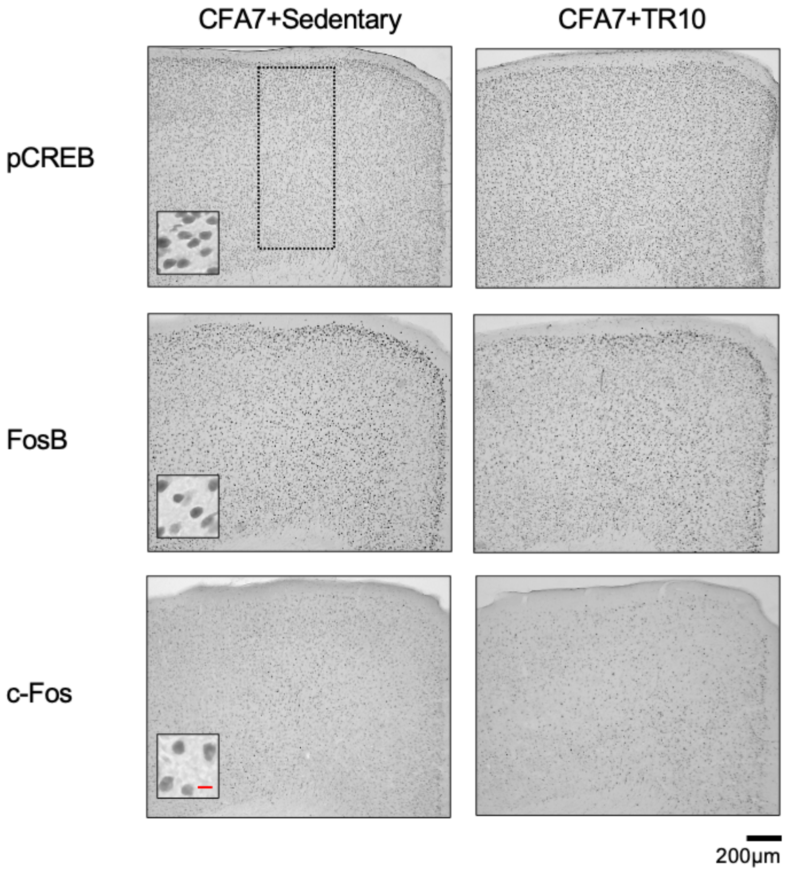

Figure S4. Microphotographs displaying pCREB, FosB, and c-Fos expression in the primary motor cortex (M1). Representative immunohistochemical images of pCREB, FosB, and c-Fos expression in the M1 (ipsilateral to CFA) in CFA-treated mice under sedentary or TR10 conditions at Day +7. These immunoreactivities were predominantly localized within cell nuclei. Cell counts for all markers were quantified within the same areas delineated by the boxes shown in the pCREB images. The scale bar in the insets represents 10 μm.

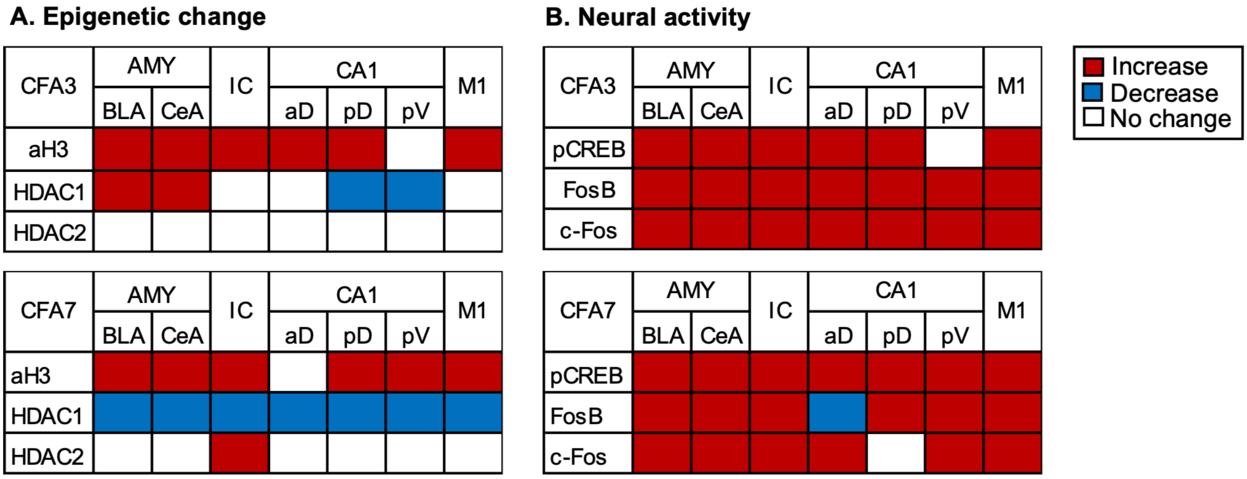

Figure S5. Summary diagram illustrating the effects of CFA-induced craniofacial inflammation on epigenetic changes and neural activity markers in the brain under sedentary conditions. This schematic summarizes significant ipsilateral changes in marker expression observed 3 or 7 days after CFA injection, expressed relative to the corresponding non-CFA sedentary groups. Red and blue shading indicate a significant increase or decrease, respectively, while blank boxes indicate no significant change compared with the corresponding non-CFA sedentary groups. Abbreviations: aH3, histone H3 acetylation; AMY, amygdala; BLA, basolateral amygdala; CeA, central amygdala; IC, insular cortex; aD, anterior dorsal CA1; pD, posterior dorsal CA1; pV, posterior ventral CA1; M1, primary motor cortex; CFA, complete Freund's adjuvant.
