# Supplementary material for: Repeated Treadmill Run Preconditioning Induces Prolonged Attenuation of Craniofacial Pain-like Behaviors and Changes in Brain Responses Associated with Persistent Craniofacial Inflammation in Male Mice
Source: Biomedicines. 2026 Jul 14;14(7):1576. doi: 10.3390/biomedicines14071576 (PMC13407325; doi:10.3390/biomedicines14071576)
Supplement: Supplementary file 1 [file biomedicines-14-01576-s001.zip › Supplementary Results R0708am9.pdf]

Supplementary Results Detailed results of the effects of CFA-induced craniofacial inflammation under sedentary conditions

Effects of CFA-induced craniofacial inflammation on epigenetic- and neural activity-related markers in each evaluated brain region. An integrated summary of these CFA-induced changes is presented in Supplementary Figure S5.

1.1. Amygdala

a. Histone H3 acetylation

Under sedentary conditions, the number of histone H3 acetylation–positive cells in both the BLA (Figure 6A) and CeA (Figure 6B) on both sides was significantly higher in the CFA3 and CFA7 groups than in the corresponding non-CFA groups (all, $p < 0.0001$).

b. HDAC1

Under sedentary conditions, HDAC1-positive cell numbers showed opposite changes between the CFA3 and CFA7 groups in both the BLA (Figure 6A) and CeA (Figure 6B). In the CFA3 group, the number of HDAC1-positive cells was significantly higher than that in the corresponding non-CFA3 group. In contrast, in the CFA7 group, the number of HDAC1-positive cells was significantly lower than that in the corresponding non-CFA7 group.

c. HDAC2

Under sedentary conditions, the number of HDAC2–positive cells was not significantly changed in the CFA3 and CFA7 groups compared with the corresponding non-CFA group in both BLA (Figure 6A) and CeA (Figure 6B).

d. pCREB

Under sedentary conditions, the number of pCREB–positive cells in the bilateral BLA and CeA in the CFA3 and CFA7 groups was significantly higher than in the corresponding non-CFA group (Figures 7A and 7B).

e. FosB

Under sedentary conditions, the number of FosB–positive cells in the bilateral BLA and CeA in the CFA3 and CFA7 groups was significantly higher than in the corresponding non-CFA group (Figures 7A and 7B).

f. c-Fos

Under sedentary conditions, the number of c-Fos–positive cells in the bilateral BLA and CeA in the CFA3 and CFA7 groups was significantly higher than in the corresponding non-CFA group (Figures 7A and 7B).

1.2. Insular cortex

a. Histone H3 acetylation

Under sedentary conditions, the number of histone H3 acetylation–positive cells in the IC on both sides was significantly higher in the CFA3 and CFA7 groups than in the corresponding non-CFA groups (Figure 9A).

b. HDAC1

Under sedentary conditions, the number of HDAC1-positive cells in the CFA7, but not the CFA3 group, was significantly lower than that in the corresponding non-CFA group (Figure 9A).

c. HDAC2

Under sedentary conditions, the number of HDAC2-positive cells in the CFA7, but not the CFA3, group was significantly higher than that in the corresponding non-CFA group (Figure 9A).

d. pCREB

Under sedentary conditions, the number of pCREB-positive cells in the CFA3 and CFA7 groups was significantly higher than that in the corresponding non-CFA group (Figure 9B).

e. FosB

Under sedentary conditions, the number of FosB-positive cells in the CFA3 and CFA7 groups was significantly higher than that in the corresponding non-CFA group (Figure 9B).

f. c-Fos

Under sedentary conditions, the number of c-Fos-positive cells in the CFA3 and CFA7 groups was significantly higher than that in the corresponding non-CFA group (Figure 9B).

1.3. Hippocampal CA1

a. Histone H3 acetylation

1. Anterior dorsal CA1 (Figure 11A)

Under sedentary conditions, the number of histone H3 acetylation–positive cells in the CFA3, but not CFA7, group was significantly higher than that in the corresponding non-CFA groups (both sides, $p < 0.0001$).

2. Posterior dorsal CA1 (Figure 11B)

Under sedentary conditions, the number of histone H3 acetylation–positive cells in the CFA3 and CFA7 groups was significantly higher than that in the corresponding non-CFA groups.

3. Posterior ventral CA1 (Figure 11C)

Under sedentary conditions, the number of histone H3 acetylation–positive cells in the CFA3 and CFA7 groups was significantly higher than that in the corresponding non-CFA groups on both sides, except for the ipsilateral side in the CFA3 group.

b. HDAC1

1. Anterior dorsal CA1 (Figure 11A)

Under sedentary conditions, the number of HDAC1-positive cells on the ipsilateral and contralateral sides was significantly lower in the CFA7 group than in the non-CFA7 group ($p < 0.0001$).

2. Posterior dorsal CA1 (Figure 11B)

Under sedentary conditions, the number of HDAC1-positive cells on the ipsilateral and contralateral sides was significantly lower in the CFA3 ($p < 0.05$) and CFA7 ($p < 0.0001$) groups than in the corresponding non-CFA group.

3. Posterior ventral CA1 (Figure 11C)

Under sedentary conditions, the number of HDAC1-positive cells on both sides was significantly lower in the CFA3 ($p < 0.05$ for the ipsilateral side, $p < 0.001$ for the contralateral side) and CFA7 ($p < 0.0001$ for the ipsilateral side, $p < 0.001$ for the contralateral side) groups than in the corresponding non-CFA group.

c. HDAC2

Under sedentary conditions, the number of HDAC2-positive cells in the three CA1 subregions did not significantly differ between the CFA and corresponding non-CFA groups.

d. pCREB

1. Anterior dorsal CA1 (Figure 12A)

Under sedentary conditions, the number of pCREB-positive cells on both sides was significantly higher in the CFA3 ($p < 0.001$ for the ipsilateral side, $p < 0.0001$ for the contralateral side) and CFA7 (both sides, $p < 0.05$) groups than in the corresponding non-CFA group.

2. Posterior dorsal CA1 (Figure 12B)

Under sedentary conditions, the number of pCREB-positive cells on both sides was significantly higher in the CFA3 (both sides, $p < 0.0001$) and CFA7 (both sides, $p < 0.0001$) groups than in the corresponding non-CFA group.

3. Posterior ventral CA1 (Figure 12C)

Under sedentary conditions, the number of pCREB-positive cells on both sides was significantly higher in the CFA7 (all, $p < 0.0001$), but not in the CFA3, group than in the corresponding non-CFA group.

e. FosB

1. Anterior dorsal CA1 (Figure 12A)

Under sedentary conditions, the number of FosB-positive cells on the ipsilateral ($p < 0.001$) and contralateral ($p < 0.05$) sides was significantly higher in the CFA3 group than in the corresponding non-CFA group. In contrast, the number of positive cells on both sides in the CFA7 group was significantly lower than the corresponding non-CFA7 group ($p < 0.05$).

2. Posterior dorsal CA1 (Figure 12B)

Under sedentary conditions, the number of FosB-positive cells on both sides was significantly higher in both CFA3 and CFA7 groups than in the corresponding non-CFA group ($p < 0.05$ for ipsilateral sides, $p < 0.0001$ for contralateral sides).

3. Posterior ventral CA1 (Figure 12C)

Under sedentary conditions, the number of FosB-positive cells on both sides was significantly higher in both CFA3 and CFA7 groups than in the corresponding non-CFA group ($p < 0.0001$).

f. c-Fos

1. Anterior dorsal CA1 (Figure 12A)

Under sedentary conditions, the number of c-Fos-positive cells on both sides (all, $p < 0.0001$) was significantly higher in the CFA3 and CFA7 groups than in the corresponding non-CFA group.

2. Posterior dorsal CA1 (Figure 12B)

Under sedentary conditions, the number of c-Fos-positive cells on the ipsilateral side ($p < 0.0001$) and contralateral side ($p < 0.05$) was significantly higher in the CFA3 than the corresponding non-CFA group.

3. Posterior ventral CA1 (Figure 12C)

Under sedentary conditions, the number of c-Fos-positive cells on the ipsilateral ($p < 0.0001$ for CFA3, $p < 0.001$ for CFA7) and contralateral ($p < 0.0001$ for both CFA3 and CFA7 groups) sides was significantly higher in the CFA3 and CFA7 groups than in the corresponding non-CFA group.

1.4. Primary motor cortex (M1)

a. Histone H3 acetylation

Under sedentary conditions, the number of histone H3 acetylation–positive cells in the bilateral M1 in the CFA3 and CFA7 groups was significantly higher than that in the corresponding non-CFA groups (Figure 14A).

b. HDAC1

Under sedentary conditions, the number of HDAC1-positive cells in the M1 on the contralateral side was significantly higher in the CFA3 group than in the corresponding non-CFA groups ($p < 0.05$). In contrast, in the CFA7 group, the number of HDAC1-positive cells was significantly lower than that in the corresponding non-CFA group (both sides, $p < 0.0001$).

c. HDAC2

Under sedentary conditions, the number of HDAC2–positive cells in the bilateral M1 did not significantly differ between the CFA3 or CFA7 groups and the corresponding non-CFA groups (Figure 14A).

d. pCREB

Under sedentary conditions, the number of pCREB–positive cells in the bilateral M1 in the CFA3 ($p < 0.0001$) and CFA7 ($p < 0.05$) groups was significantly higher than that in the corresponding non-CFA groups (Figure 14B).

e. FosB

Under sedentary conditions, the number of FosB–positive cells in the bilateral M1 was significantly higher in the CFA3 and CFA7 groups than in the corresponding non-CFA groups (Figure 14B).

f. c-Fos

Under sedentary conditions, the number of c-Fos–positive cells in the bilateral M1 was significantly higher in the CFA3 and CFA7 groups than in the corresponding non-CFA groups (Figure 14B).
