# Supplementary material for: Repeated Treadmill Run Preconditioning Induces Prolonged Attenuation of Craniofacial Pain-like Behaviors and Changes in Brain Responses Associated with Persistent Craniofacial Inflammation in Male Mice
Source: Biomedicines. 2026 Jul 14;14(7):1576. doi: 10.3390/biomedicines14071576 (PMC13407325; doi:10.3390/biomedicines14071576)
Supplement: Supplementary file 1 [file biomedicines-14-01576-s001.zip › Table S1 Sample size 0617.pdf]

Supplemental Table S1. Details on number of animals used in the present study

A. Sample size used in the behavioral tests and lactate assay

A total of 90 mice were used for the assessment of anxiety-like behavior test using the elevated plus maze (EPM) and open field (OF) tests. In a separate experiment (*), 80 mice were employed to assess orofacial pain-like behavior test (orofacial formalin test, F-test), and 16 mice were employed to assess brain lactate level.

EPM	Non-CFA3	Non-CFA7	CFA3	CFA7	Total
**Sedentary**	10	10	10	10	40
**TR10**	11	10	10	9	40
**Total**	**21**	**20**	**20**	**19**	**80**

OF	Non-CFA3	Non-CFA7	CFA3	CFA7	Total
**Sedentary**	13	13	10	10	46
**TR10**	15	10	10	9	44
**Total**	**28**	**23**	**20**	**19**	**90**

F-test*	Non-CFA3	Non-CFA7	CFA3	CFA7	Total
**Sedentary**	10	10	10	10	40
**TR10**	10	10	10	10	40
**Total**	**20**	**20**	**20**	**20**	**80**

Brain lactate*	Non-CFA3	Non-CFA7	CFA3	CFA7	Total
**Sedentary**	2	2	2	2	8
**TR10**	2	2	2	2	8
**Total**	**4**	**4**	**4**	**4**	**16**

B. The sample size used for the quantification of immuno-positive cells for each marker in each brain region.

Amygdala

Histone H3 acetylation

BLA	Non-CFA3	Non-CFA7	CFA3	CFA7
**Sedentary**	7	7	10	10
**TR10**	10	10	9	9
**Total**	**17**	**17**	**19**	**19**

HDAC1

BLA	Non-CFA3	Non-CFA7	CFA3	CFA7
**Sedentary**	7	7	6	6
**TR10**	7	9	7	7
**Total**	**14**	**16**	**13**	**13**

HDAC2

BLA	Non-CFA3	Non-CFA7	CFA3	CFA7
**Sedentary**	7	7	6	6
**TR10**	7	9	7	7
**Total**	**14**	**16**	**13**	**13**

pCREB

BLA	Non-CFA3	Non-CFA7	CFA3	CFA7
**Sedentary**	7	7	10	10
**TR10**	10	10	10	9
**Total**	**17**	**17**	**20**	**19**

FosB

BLA	Non-CFA3	Non-CFA7	CFA3	CFA7
**Sedentary**	7	7	10	10
**TR10**	10	10	10	9
**Total**	**17**	**17**	**20**	**19**

c-Fos

BLA	Non-CFA3	Non-CFA7	CFA3	CFA7
**Sedentary**	7	7	6	10
**TR10**	10	10	7	9
**Total**	**17**	**17**	**13**	**19**

Histone H3 acetylation

CeA	Non-CFA3	Non-CFA7	CFA3	CFA7
**Sedentary**	7	7	10	10
**TR10**	10	10	9	9
**Total**	**17**	**17**	**19**	**19**

HDAC1

CeA	Non-CFA3	Non-CFA7	CFA3	CFA7
**Sedentary**	7	7	6	6
**TR10**	7	9	7	6
**Total**	**14**	**16**	**13**	**12**

HDAC2

CeA	Non-CFA3	Non-CFA7	CFA3	CFA7
**Sedentary**	7	7	6	6
**TR10**	7	9	7	7
**Total**	**14**	**16**	**13**	**13**

pCREB

CeA	Non-CFA3	Non-CFA7	CFA3	CFA7
**Sedentary**	7	7	10	10
**TR10**	10	10	10	9
**Total**	**17**	**17**	**20**	**19**

FosB

CeA	Non-CFA3	Non-CFA7	CFA3	CFA7
Sedentary	7	7	10	10
TR10	10	10	10	9
**Total**	**17**	**17**	**20**	**19**

c-Fos

CeA	Non-CFA3	Non-CFA7	CFA3	CFA7
Sedentary	7	7	6	10
TR10	10	10	7	9
**Total**	**17**	**17**	**13**	**19**

Abbreviations; BLA, basolateral subregion; CeA, central subregion

Insular Cortex (IC)

Histone H3 acetylation

IC	Non-CFA3	Non-CFA7	CFA3	CFA7
Sedentary	7	7	10	10
TR10	9	10	8	9
**Total**	**16**	**17**	**18**	**19**

HDAC1

IC	Non-CFA3	Non-CFA7	CFA3	CFA7
Sedentary	7	7	6	6
TR10	6	10	7	6
**Total**	**13**	**17**	**13**	**12**

HDAC2

IC	Non-CFA3	Non-CFA7	CFA3	CFA7
Sedentary	7	7	6	6
TR10	6	10	7	6
**Total**	**13**	**17**	**13**	**12**

pCREB

IC	Non-CFA3	Non-CFA7	CFA3	CFA7
Sedentary	7	7	9	10
TR10	10	10	10	9
**Total**	**17**	**17**	**19**	**19**

FosB

IC	Non-CFA3	Non-CFA7	CFA3	CFA7
Sedentary	7	7	9	10
TR10	9	10	9	9
**Total**	**16**	**17**	**18**	**19**

c-Fos

IC	Non-CFA3	Non-CFA7	CFA3	CFA7
Sedentary	7	7	7	10
TR10	9	9	8	9
**Total**	**16**	**16**	**15**	**19**

CA1

Histone H3 acetylation

Anterior dorsal	Non-CFA3	Non-CFA7	CFA3	CFA7
**Sedentary**	7	7	7	10
**TR10**	10	10	10	9
**Total**	**17**	**17**	**17**	**19**

HDAC1

Anterior dorsal	Non-CFA3	Non-CFA7	CFA3	CFA7
**Sedentary**	7	7	6	6
**TR10**	7	10	7	6
**Total**	**14**	**17**	**13**	**12**

HDAC2

Anterior dorsal	Non-CFA3	Non-CFA7	CFA3	CFA7
**Sedentary**	7	7	6	6
**TR10**	7	10	7	6
**Total**	**14**	**17**	**13**	**12**

pCREB

Anterior dorsal	Non-CFA3	Non-CFA7	CFA3	CFA7
**Sedentary**	7	7	10	10
**TR10**	10	10	10	9
**Total**	**17**	**17**	**20**	**19**

FosB

Anterior dorsal	Non-CFA3	Non-CFA7	CFA3	CFA7
**Sedentary**	7	7	10	10
**TR10**	10	10	10	9
**Total**	**17**	**17**	**20**	**19**

c-Fos

Anterior dorsal	Non-CFA3	Non-CFA7	CFA3	CFA7
**Sedentary**	6	6	7	10
**TR10**	10	10	7	9
**Total**	**16**	**16**	**14**	**19**

Histone H3 acetylation

Posterior dorsal	Non-CFA3	Non-CFA7	CFA3	CFA7
**Sedentary**	6	6	8	10
**TR10**	10	10	10	9
**Total**	**16**	**16**	**18**	**19**

HDAC1

Posterior dorsal	Non-CFA3	Non-CFA7	CFA3	CFA7
**Sedentary**	7	7	6	6
**TR10**	7	10	7	6
**Total**	**14**	**17**	**13**	**12**

HDAC2

Posterior dorsal	Non-CFA3	Non-CFA7	CFA3	CFA7
**Sedentary**	7	7	6	6
**TR10**	7	10	7	6
**Total**	**14**	**17**	**13**	**12**

pCREB

Posterior dorsal	Non-CFA3	Non-CFA7	CFA3	CFA7
**Sedentary**	7	7	10	10
**TR10**	10	10	10	9
**Total**	**17**	**17**	**20**	**19**

FosB

Posterior dorsal	Non-CFA3	Non-CFA7	CFA3	CFA7
**Sedentary**	7	7	10	10
**TR10**	10	10	10	9
**Total**	**17**	**17**	**20**	**19**

c-Fos

Posterior dorsal	Non-CFA3	Non-CFA7	CFA3	CFA7
**Sedentary**	6	6	7	10
**TR10**	10	10	7	9
**Total**	**16**	**16**	**14**	**19**

Histone H3 acetylation

Posterior ventral	Non-CFA3	Non-CFA7	CFA3	CFA7
**Sedentary**	7	7	8	10
**TR10**	10	10	10	9
**Total**	**17**	**17**	**18**	**19**

HDAC1

Posterior ventral	Non-CFA3	Non-CFA7	CFA3	CFA7
**Sedentary**	7	7	6	6
**TR10**	7	10	7	6
**Total**	**14**	**17**	**13**	**12**

HDAC2

Posterior ventral	Non-CFA3	Non-CFA7	CFA3	CFA7
**Sedentary**	7	7	6	6
**TR10**	7	10	7	6
**Total**	**14**	**17**	**13**	**12**

pCREB

Posterior ventral	Non-CFA3	Non-CFA7	CFA3	CFA7
**Sedentary**	7	7	10	10
**TR10**	10	10	10	9
**Total**	**17**	**17**	**20**	**19**

FosB

Posterior ventral	Non-CFA3	Non-CFA7	CFA3	CFA7
**Sedentary**	7	7	10	10
**TR10**	10	10	9	9
**Total**	**17**	**17**	**19**	**19**

c-Fos

Posterior ventral	Non-CFA3	Non-CFA7	CFA3	CFA7
**Sedentary**	6	6	5	10
**TR10**	10	10	6	9
**Total**	**16**	**16**	**11**	**19**

Primary motor cortex (M1)

Histone H3 acetylation

M1	Non-CFA3	Non-CFA7	CFA3	CFA7
**Sedentary**	7	7	10	10
**TR10**	9	10	8	9
**Total**	**16**	**17**	**18**	**19**

HDAC1

M1	Non-CFA3	Non-CFA7	CFA3	CFA7
**Sedentary**	7	7	6	6
**TR10**	7	9	7	6
**Total**	**14**	**16**	**13**	**12**

HDAC2

M1	Non-CFA3	Non-CFA7	CFA3	CFA7
**Sedentary**	7	7	6	6
**TR10**	7	9	7	6
**Total**	**14**	**16**	**13**	**12**

pCREB

M1	Non-CFA3	Non-CFA7	CFA3	CFA7
**Sedentary**	7	7	9	10
**TR10**	10	10	10	9
**Total**	**17**	**17**	**19**	**19**

FosB

M1	Non-CFA3	Non-CFA7	CFA3	CFA7
**Sedentary**	7	7	9	10
**TR10**	9	10	9	9
**Total**	**16**	**17**	**18**	**19**

c-Fos

M1	Non-CFA3	Non-CFA7	CFA3	CFA7
**Sedentary**	7	7	8	10
**TR10**	9	10	7	9
**Total**	**16**	**17**	**15**	**19**

C. The sample size used for Spearman's correlation analysis

Histone H3 acetylation	Non-CFA3	Non-CFA7	CFA3	CFA7
**Sedentary**	7	6	7	10
**TR10**	9	10	8	9
**Total**	**16**	**16**	**15**	**19**

pCREB	Non-CFA3	Non-CFA7	CFA3	CFA7
**Sedentary**	7	7	9	10
**TR10**	10	10	10	9
**Total**	**17**	**17**	**19**	**19**

FosB	Non-CFA3	Non-CFA7	CFA3	CFA7
**Sedentary**	7	7	9	10
**TR10**	9	10	9	9
**Total**	**16**	**17**	**18**	**19**

c-Fos	Non-CFA3	Non-CFA7	CFA3	CFA7
**Sedentary**	6	6	5	10
**TR10**	9	9	7	9
**Total**	**15**	**15**	**12**	**19**
