# Supplementary material for: Repeated Treadmill Run Preconditioning Induces Prolonged Attenuation of Craniofacial Pain-like Behaviors and Changes in Brain Responses Associated with Persistent Craniofacial Inflammation in Male Mice
Source: Biomedicines. 2026 Jul 14;14(7):1576. doi: 10.3390/biomedicines14071576 (PMC13407325; doi:10.3390/biomedicines14071576)
Supplement: Supplementary file 1 [file biomedicines-14-01576-s001.zip › Table S2 Materials 0617.pdf]

**Supplemental Table S2. Key resources used in this study.**
This table summarizes the major reagents, antibodies, solutions, and equipment employed in the experiments, grouped by category, along with supplier details (company, city, country) to facilitate reproducibility.

**A. Chemicals and Solutions**

| Reagent/Material | Source/Supplier (Company, City, Country) |
|---|---|
| Complete Freund's Adjuvant (CFA) | Sigma-Aldrich, St. Louis, MO, USA |
| 10% Formalin solution | Fujifilm-Wako, Osaka, Japan |
| Anesthetic agents (3-Mixed solution):<br><br>- Medetomidine | <br><br>Domitor; Nippon Zen-Yaku Kogyo, Koriyama, Fukushima, Japan |
| - Midazolam | Midazolam; Sandoz, Kamiyama, Yamagata, Japan |
| - Butorphanol | Vetorphale; Meiji Seika Pharma, Tokyo, Japan |
| Paraformaldehyde (PFA) | Wako, Osaka, Japan |
| Sucrose | Wako, Osaka, Japan |
| Normal Goat Serum (NGS) | Jackson Immuno Research Laboratories Inc. West Grove, PA, USA |
| Diaminobenzidine | Tokyo Chemical Industry, Tokyo, Japan |
| Ammonium Nickel (II) Sulfate Hexahydrate | Wako, Osaka, Japan |
| Peroxidase ($H_2O_2$) | Fujifilm-Wako, Osaka, Japan |
| Avidin-Biotin Complex Staining Kits (ABC Kits) | Vector Laboratories, Newark, CA, USA |
| Lactate Assay Kit-WST | DOJINDO LABORATORIES, Kumamoto, Japan; Cat. No. L256 |

**B. Antibodies**

| Reagent/Material | Source/Supplier (Company, Cat#, Research Resource Identifiers (RRID), City, Country) |
|---|---|
| FosB monoclonal antibody | Abcam, ab184938, AB_2721123, Cambridge, UK |
| c-Fos polyclonal antibody | Abcam, 2250S, AB_2247211, Cambridge, UK |
| Phospho-CREB polyclonal antibody | Sigma-Aldrich, 06-519, AB_310153, Massachusetts, USA |
| Affinity-purified rabbit histone H3 acetylation polyclonal antibody | Sigma-Aldrich, 06-599, AB_3683677, Massachusetts, USA |
| HDAC1 polyclonal antibody | Abcam, ab19845, AB_470299, Cambridge, UK |
| HDAC2 monoclonal antibody | Abcam, ab32117, AB_732777, Cambridge, UK |
| Biotinylated goat anti-rabbit IgG antibody | Vector Laboratories, BA-1000, AB_2313606, Burlingame, CA, USA |

**C. Equipment**

| Reagent/Material | Source/Supplier (Company, City, Country) |
|---|---|
| Treadmill apparatus | TMS-2B, MELQUEST Co., Ltd., Toyama, Japan |
| Freezing microtome | REM-710, RETRATOME, YAMATO, Saitama, Japan |
| Elevated plus-maze (EPM) apparatus | PM-DR25M-S, Brain Science Idea. Co., Ltd., Osaka, Japan |
| SCANET MV-40 MOV | MEL-QUEST Co., LTD., Toyama, Japan |
| Light microscope | EVOS XL Core, Thermo Fisher Scientific, Waltham, MA, USA |
| Microplate reader | Multiskan FC, ThermoFisher Scientific, Waltham, MA, USA |
