# Supplementary material for: Repeated Treadmill Run Preconditioning Induces Prolonged Attenuation of Craniofacial Pain-like Behaviors and Changes in Brain Responses Associated with Persistent Craniofacial Inflammation in Male Mice
Source: Biomedicines. 2026 Jul 14;14(7):1576. doi: 10.3390/biomedicines14071576 (PMC13407325; doi:10.3390/biomedicines14071576)
Supplement: Supplementary file 1 [file biomedicines-14-01576-s001.zip › Table S3 Main effect 0617.pdf]

Supplemental Table S3. Statistical data for the main effects for between-subject factors (group comparisons) and within-subject factors (ipsilateral versus contralateral sides) in the CFA3 group.

CFA 3	AMY		IC	CA1			M1
	BLA	CeA		aD	pD	pV	
**aH3**							
- Between groups	$F(3, 33) = 19.2$, $p =$ **0.0001***	$F(3, 32) = 27.7$, $p =$ **0.0001***	$F(3, 30) = 7.42$, $p =$ **0.001**	$F(3, 30) = 36.9$, $p =$ **0.0001***	$F(3, 30) = 17.04$, $p =$ **0.0001***	$F(3, 31) = 8.62$, $p =$ **0.0001***	$F(3, 30) = 5.12$, $p =$ **0.006*
- Laterality	$F(1, 33) = 0.36$, $p = 0.552$	$F(1, 32) = 1.26$, $p = 0.271$	$F(1, 30) = 0.13$, $p = 0.717$	$F(1, 30) = 0.42$, $p = 0.840$	$F(1, 30) = 0.54$, $p = 0.466$	$F(1, 31) = 1.78$, $p = 0.192$	$F(1, 30) = 1.29$, $p = 0.265$
**HDAC1**							
- Between groups	$F(3, 23) = 6.4$, $p =$ **0.03*	$F(3, 23) = 15.1$, $p =$ **0.0001***	$F(3, 22) = 2.62$, $p = 0.076$	$F(3, 23) = 20.7$, $p =$ **0.0001***	$F(3, 23) = 6.67$, $p =$ **0.002*	$F(3, 23) = 11.7$, $p =$ **0.0001***	$F(3, 23) = 6.76$, $p =$ **0.002*
- Laterality	$F(1, 23) = 0.87$, $p = 0.77$	$F(1, 23) = 2.34$, $p = 0.138$	$F(1, 22) = 2.93$, $p = 0.101$	$F(1, 23) = 0.02$, $p = 0.878$	$F(1, 23) = 0.03$, $p = 0.894$	$F(1, 23) = 0.007$, $p = 0.935$	$F(1, 23) = 2.12$, $p = 0.159$
**HDAC2**							
- Between groups	$F(3, 23) = 8.68$, $p =$ **0.0001***	$F(3, 23) = 13.8$, $p =$ **0.0001***	$F(3, 22) = 3.22$, $p = 0.357$	$F(3, 23) = 4.18$, $p = 0.17$	$F(3, 23) = 2.92$, $p = 0.056$	$F(3, 23) = 2.26$, $p = 0.088$	$F(3, 23) = 2.46$, $p = 0.089$
- Laterality	$F(1, 23) = 0.36$, $p = 0.556$	$F(1, 23) = 1.75$, $p = 0.20$	$F(1, 22) = 0.01$, $p = 0.937$	$F(1, 23) = 0.15$, $p = 0.7$	$F(1, 23) = 0.001$, $p = 0.98$	$F(1, 23) = 6.64$, $p =$ **0.017*	$F(1, 23) = 0.74$, $p = 0.399$
**pCREB**							
- Between groups	$F(3, 32) = 22.2$, $p =$ **0.0001***	$F(3, 33) = 32.0$, $p =$ **0.0001***	$F(3, 32) = 67.9$, $p =$ **0.0001***	$F(3, 33) = 11.4$, $p =$ **0.0001***	$F(3, 33) = 32.5$, $p =$ **0.0001***	$F(3, 33) = 4.69$, $p =$ **0.008*	$F(3, 32) = 15.3$, $p =$ **0.0001***
- Laterality	$F(1, 32) = 0.19$, $p = 0.99$	$F(1, 33) = 0.67$, $p = 0.419$	$F(1, 32) = 0.03$, $p = 0.858$	$F(1, 33) = 0.20$, $p = 0.654$	$F(1, 33) = 0.01$, $p = 0.921$	$F(1, 33) = 0.001$, $p = 0.972$	$F(1, 32) = 1.85$, $p = 0.183$
**FosB**							
- Between groups	$F(3, 33) = 19.2$, $p =$ **0.0001***	$F(3, 33) = 33.1$, $p =$ **0.0001***	$F(3, 30) = 175.1$, $p =$ **0.0001***	$F(3, 33) = 10.4$, $p =$ **0.0001***	$F(3, 33) = 16.5$, $p =$ **0.0001***	$F(3, 32) = 48.9$, $p =$ **0.0001***	$F(3, 30) = 25.9$, $p =$ **0.0001***
- Laterality	$F(1, 33) = 0.36$, $p = 0.552$	$F(1, 33) = 0.52$, $p = 0.477$	$F(1, 30) = 0.45$, $p = 0.509$	$F(1, 33) = 0.001$, $p = 0.972$	$F(1, 33) = 0.01$, $p = 0.936$	$F(1, 32) = 0.32$, $p = 0.575$	$F(1, 30) = 0.02$, $p = 0.876$
**c-Fos**							
- Between groups	$F(3, 26) = 22.6$, $p =$ **0.0001***	$F(3, 26) = 57.9$, $p =$ **0.0001***	$F(3, 27) = 22.3$, $p =$ **0.0001***	$F(3, 26) = 43.8$, $p =$ **0.0001***	$F(3, 26) = 10.7$, $p =$ **0.0001***	$F(3, 22) = 16.8$, $p =$ **0.0001***	$F(3, 27) = 5.42$, $p =$ **0.005*
- Laterality	$F(1, 26) = 0.49$, $p = 0.489$	$F(1, 26) = 1.57$, $p = 0.221$	$F(1, 27) = 0.20$, $p = 0.657$	$F(1, 26) = 0.51$, $p = 0.481$	$F(1, 26) = 16.6$, $p =$ **0.0001***	$F(1, 22) = 0.37$, $p = 0.547$	$F(1, 27) = 1.45$, $p = 0.24$

*, $p < 0.05$; **, $p < 0.001$; ***, $p < 0.0001$. Abbreviations: aD, anterior dorsal CA1; aH3, histone H3 acetylation; AMY, amygdala; BLA, basolateral amygdala; CA1, hippocampal CA1 region; CeA, central amygdala; CFA, complete Freund's adjuvant; HDAC1, histone deacetylase 1; HDAC2, histone deacetylase 2; IC, insular cortex; M1, primary motor cortex; pD, posterior dorsal CA1; pV, posterior ventral CA1.
