# Supplementary material for: Repeated Treadmill Run Preconditioning Induces Prolonged Attenuation of Craniofacial Pain-like Behaviors and Changes in Brain Responses Associated with Persistent Craniofacial Inflammation in Male Mice
Source: Biomedicines. 2026 Jul 14;14(7):1576. doi: 10.3390/biomedicines14071576 (PMC13407325; doi:10.3390/biomedicines14071576)
Supplement: Supplementary file 1 [file biomedicines-14-01576-s001.zip › Table S4 Main effect 0617.pdf]

Supplemental Table S4. Statistical data for the main effects for between-subject factors (group comparisons) and within-subject factors (ipsilateral versus contralateral sides) in the CFA7 group.

CFA 7	AMY		IC	CA1			M1
	BLA	CeA		aD	pD	pV	
**aH3**							
- Between groups	$F (3, 32) = 60.15$, $p = \mathbf{0.0001}^{***}$	$F (3, 32) = 106.8$, $p = \mathbf{0.0001}^{***}$	$F (3, 32) = 19.9$, $p = \mathbf{0.0001}^{***}$	$F (3, 32) = 8.36$, $p = \mathbf{0.0001}^{***}$	$F (3, 31) = 42.1$, $p = \mathbf{0.0001}^{***}$	$F (3, 32) = 22.3$, $p = \mathbf{0.0001}^{***}$	$F (3, 32) = 13.2$, $p = \mathbf{0.0001}^{***}$
- Laterality	$F (1, 32) = 0.47$, $p = 0.497$	$F (1, 32) = 0.42$, $p = 0.521$	$F (1, 32) = 2.69$, $p = 0.111$	$F (1, 32) = 0.92$, $p = 0.345$	$F (1, 31) = 0.09$, $p = 0.767$	$F (1, 32) = 2.45$, $p = 0.128$	$F (1, 32) = 2.35$, $p = 0.135$
**HDAC1**							
- Between groups	$F (3, 25) = 25.9$, $p = \mathbf{0.0001}^{***}$	$F (3, 25) = 28.1$, $p = \mathbf{0.0001}^{***}$	$F (3, 25) = 11.2$, $p = \mathbf{0.0001}^{***}$	$F (3, 25) = 12.9$, $p = \mathbf{0.0001}^{***}$	$F (3, 25) = 38.1$, $p = \mathbf{0.0001}^{***}$	$F (3, 25) = 32.8$, $p = \mathbf{0.0001}^{***}$	$F (3, 24) = 9.17$, $p = \mathbf{0.0001}^{***}$
- Laterality	$F (1, 25) = 0.2$, $p = 0.659$	$F (1, 25) = 0.78$, $p = 0.386$	$F (1, 25) = 0.28$, $p = 0.601$	$F (1, 25) = 0.16$, $p = 0.969$	$F (1, 25) = 0.50$, $p = 0.485$	$F (1, 25) = 3.93$, $p = 0.058$	$F (1, 24) = 0.13$, $p = 0.719$
**HDAC2**							
- Between groups	$F (3, 25) = 3.01$, $p = \mathbf{0.049}^{*}$	$F (3, 25) = 6.54$, $p = \mathbf{0.002}^{*}$	$F (3, 25) = 6.58$, $p = \mathbf{0.002}^{*}$	$F (3, 25) = 3.96$, $p = \mathbf{0.019}^{*}$	$F (3, 25) = 3.87$, $p = \mathbf{0.021}^{*}$	$F (3, 25) = 2.86$, $p = 0.057$	$F (3, 24) = 7.31$, $p = \mathbf{0.001}^{**}$
- Laterality	$F (1, 25) = 0.63$, $p = 0.804$	$F (1, 25) = 0.001$, $p = 0.985$	$F (1, 25) = 3.12$, $p = 0.090$	$F (1, 25) = 1.13$, $p = 0.299$	$F (1, 25) = 1.06$, $p = 0.314$	$F (1, 25) = 1.21$, $p = 0.281$	$F (1, 24) = 0.28$, $p = 0.600$
**pCREB**							
- Between groups	$F (3, 32) = 55.5$, $p = \mathbf{0.0001}^{***}$	$F (3, 32) = 22.6$, $p = \mathbf{0.0001}^{***}$	$F (3, 32) = 35.4$, $p = \mathbf{0.0001}^{***}$	$F (3, 32) = 16.0$, $p = \mathbf{0.0001}^{***}$	$F (3, 32) = 122.5$, $p = \mathbf{0.0001}^{***}$	$F (3, 32) = 77.4$, $p = \mathbf{0.0001}^{***}$	$F (3, 32) = 6.22$, $p = \mathbf{0.002}^{*}$
- Laterality	$F (1, 32) = 0.99$, $p = 0.328$	$F (1, 32) = 0.99$, $p = 0.304$	$F (1, 32) = 0.001$, $p = 0.989$	$F (1, 32) = 1.96$, $p = 0.172$	$F (1, 32) = 0.002$, $p = 0.968$	$F (1, 32) = 1.34$, $p = 0.256$	$F (1, 32) = 0.03$, $p = 0.870$
**FosB**							
- Between groups	$F (3, 32) = 34.5$, $p = \mathbf{0.0001}^{***}$	$F (3, 32) = 150.9$, $p = \mathbf{0.0001}^{***}$	$F (3, 32) = 41.5$, $p = \mathbf{0.0001}^{***}$	$F (3, 32) = 6.69$, $p = \mathbf{0.001}^{**}$	$F (3, 32) = 10.9$, $p = \mathbf{0.0001}^{***}$	$F (3, 32) = 27.6$, $p = \mathbf{0.0001}^{***}$	$F (3, 32) = 25.6$, $p = \mathbf{0.0001}^{***}$
- Laterality	$F (1, 32) = 2.34$, $p = 0.136$	$F (1, 32) = 0.01$, $p = 0.945$	$F (1, 32) = 0.19$, $p = 0.658$	$F (1, 32) = 1.45$, $p = 0.237$	$F (1, 32) = 0.13$, $p = 0.726$	$F (1, 32) = 0.003$, $p = 0.960$	$F (1, 32) = 0.02$, $p = 0.905$
**c-Fos**							
- Between groups	$F (3, 32) = 49.7$, $p = \mathbf{0.0001}^{***}$	$F (3, 32) = 136.5$, $p = \mathbf{0.0001}^{***}$	$F (3, 31) = 4.16$, $p = \mathbf{0.014}^{*}$	$F (3, 31) = 59.3$, $p = \mathbf{0.0001}^{***}$	$F (3, 31) = 4.72$, $p = \mathbf{0.008}^{*}$	$F (3, 31) = 19.2$, $p = \mathbf{0.0001}^{***}$	$F (3, 32) = 9.06$, $p = \mathbf{0.0001}^{***}$
- Laterality	$F (1, 32) = 0.27$, $p = 0.608$	$F (1, 32) = 0.01$, $p = 0.918$	$F (1, 31) = 9.47$, $p = \mathbf{0.004}^{*}$	$F (1, 31) = 0.76$, $p = 0.391$	$F (1, 31) = 10.8$, $p = \mathbf{0.002}^{*}$	$F (1, 31) = 0.13$, $p = 0.725$	$F (1, 32) = 0.37$, $p = 0.547$

*, $p < 0.05$; **, $p < 0.001$; ***, $p < 0.0001$. Abbreviations: aD, anterior dorsal CA1; aH3, histone H3 acetylation; AMY, amygdala; BLA, basolateral amygdala; CA1, hippocampal CA1 region; CeA, central amygdala; CFA, complete Freund's adjuvant; HDAC1, histone deacetylase 1; HDAC2, histone deacetylase 2; IC, insular cortex; M1, primary motor cortex; pD, posterior dorsal CA1; pV, posterior ventral CA1.
